# Nutrient and drought stress: implications for phenology and biomass quality in miscanthus

**DOI:** 10.1093/aob/mcy155

**Published:** 2018-08-21

**Authors:** Ricardo M F da Costa, Rachael Simister, Luned A Roberts, Emma Timms-Taravella, Arthur B Cambler, Fiona M K Corke, Jiwan Han, Richard J Ward, Marcos S Buckeridge, Leonardo D Gomez, Maurice Bosch

**Affiliations:** 1 Institute of Biological, Environmental and Rural Sciences (IBERS), Aberystwyth University, Plas Gogerddan, Aberystwyth, UK; 2 CNAP, Department of Biology, University of York, Heslington, York, UK; 3 The National Plant Phenomics Centre, Institute of Biological, Environmental and Rural Sciences, Aberystwyth University, Aberystwyth, UK; 4 Department of Chemistry, Faculdade de Filosofia, Ciências e Letras de Ribeirão Preto, University of São Paulo, Ribeirão Preto-SP, Brazil; 5 Department of Botany, Institute of Biosciences, University of São Paulo, São Paulo, Brazil

**Keywords:** Bioenergy, biomass quality, cell wall, drought stress, environmental conditions, growth and development, marginal land, miscanthus, nutrient stress, phenotyping, recalcitrance, sugar release

## Abstract

**Background and Aims:**

The cultivation of dedicated biomass crops, including miscanthus, on marginal land provides a promising approach to the reduction of dependency on fossil fuels. However, little is known about the impact of environmental stresses often experienced on lower-grade agricultural land on cell-wall quality traits in miscanthus biomass crops. In this study, three different miscanthus genotypes were exposed to drought stress and nutrient stress, both separately and in combination, with the aim of evaluating their impact on plant growth and cell-wall properties.

**Methods:**

Automated imaging facilities at the National Plant Phenomics Centre (NPPC-Aberystwyth) were used for dynamic phenotyping to identify plant responses to separate and combinatorial stresses. Harvested leaf and stem samples of the three miscanthus genotypes (*Miscanthus sinensis*, *Miscanthus sacchariflorus* and *Miscanthus × giganteus*) were separately subjected to saccharification assays, to measure sugar release, and cell-wall composition analyses.

**Key Results:**

Phenotyping showed that the *M. sacchariflorus* genotype Sac-5 and particularly the *M. sinensis* genotype Sin-11 coped better than the *M. × giganteus* genotype Gig-311 with drought stress when grown in nutrient-poor compost. Sugar release by enzymatic hydrolysis, used as a biomass quality measure, was significantly affected by the different environmental conditions in a stress-, genotype- and organ-dependent manner. A combination of abundant water and low nutrients resulted in the highest sugar release from leaves, while for stems this was generally associated with the combination of drought and nutrient-rich conditions. Cell-wall composition analyses suggest that changes in fine structure of cell-wall polysaccharides, including heteroxylans and pectins, possibly in association with lignin, contribute to the observed differences in cell-wall biomass sugar release.

**Conclusions:**

The results highlight the importance of the assessment of miscanthus biomass quality measures in addition to biomass yield determinations and the requirement for selecting suitable miscanthus genotypes for different environmental conditions.

## INTRODUCTION

There is an urgent need for further deployment of renewable energy options to reduce dependency on fossil fuels, which represent a finite resource and cause environmental damage. Plant biomass is a promising renewable resource to achieve a low-carbon bio-economy with the production of biofuels, pharmaceuticals, platform chemicals and energy for heat, power and fuel. However, in some geographical regions there is intense competition for land as we are also faced with the challenge of providing food security to the world’s growing population. Using food crop residues as feedstock for biorefining could partly alleviate this competition for land use. A complementary strategy to optimize land use is to cultivate dedicated biomass crops on under-utilized lower-grade agricultural land, also referred to as marginal land. This would avoid displacement of crops currently used for food and feed production from productive agricultural land (Valentine *et al.*, 2012).

Species and hybrids of the C4 perennial rhizomatous grasses from the *Miscanthus* genus display remarkable adaptability to different environments, combining high productivity with excellent cold adaptation with a natural geographical range extending from the tropics in South-East Asia through northern China, Japan and Siberia (Donnison and Fraser, 2016). These characteristics make miscanthus one of the leading candidate crops for biomass production on marginal land (Clifton-Brown *et al.*, 2017). Moreover, the cultivation of miscanthus species on contaminated, degraded and marginal land can enhance soil quality, organic matter concentration and organism diversity whilst achieving reasonable biomass yields (Nsanganwimana *et al.*, 2014; Pidlisnyuk *et al.*, 2014; Jezowski *et al.*, 2017). However, crops growing on marginal land are subjected to a range of abiotic stresses, including water deficit and poor nutrient availability (Jones *et al.*, 2015). The full potential of miscanthus as a lignocellulose feedstock for biorefining is therefore largely dependent on biomass yield and quality when exposed to the stresses encountered when cultivated on marginal land.

The effects of different environmental conditions on crop physiology and biomass yield have been well studied in miscanthus in the case of drought stress (Ings *et al.*, 2013; Malinowska *et al.*, 2017), salt stress (Płażek *et al.*, 2014; Chen *et al.*, 2017; Stavridou *et al.*, 2017), cold stress (Purdy *et al.*, 2013; Friesen *et al.*, 2014; Głowacka *et al.*, 2014) and elevated CO_2_ (De Souza *et al.*, 2013). Most of the information on nutrient availability relates to nitrogen fertilization in experimental field trials in Europe and the USA (Davey *et al.*, 2017), in particular with a single triploid genotype of *Miscanthus × giganteus* (Behnke *et al.*, 2012; Wang *et al.*, 2012; Dierking *et al.*, 2016; Lee *et al*., 2017), a natural sterile hybrid of *M. sacchariflorus* and *M. sinensis* (Hodkinson *et al.*, 2002). The expense of using nitrogen fertilizer and its impact on greenhouse gas emissions are essential factors in determining the sustainability and financial profitability of energy crop production (Shield *et al.*, 2014).

Biomass quality parameters depend on the intended technology for lignocellulose conversion. Lignin, ash and moisture content are some of the main parameters that influence biomass quality for thermochemical conversion, such as combustion, gasification and pyrolysis (Tanger *et al*., 2013). Biochemical conversion of cell-wall polysaccharides into readily usable free sugars relies on hydrolytic enzymes. The sugars released can be fermented into alcohols, organic acids or hydrocarbons by microorganisms. In this case, the main biomass quality parameter is related to the efficiency with which enzymes can access cell-wall polysaccharides to produce free sugars (De Souza *et al.*, 2014). Identification of the factors that govern the inherent recalcitrance of cell-wall biomass to sugar release and developing strategies to overcome this recalcitrance are essential for the commercial exploitation of dedicated biomass crops, including miscanthus.

The plant cell wall is a dynamic structure that not only provides mechanical support, but also responds to various environmental and developmental cues and fulfils important functions in signalling events, defence against biotic and abiotic stresses, and growth (Bosch *et al.*, 2011). Abiotic stress-induced changes in plant cell-wall composition and architecture are important for plant adaptation and can play an important role in plant resistance to abiotic stress (Le Gall *et al.*, 2015). However, the mechanistic relationship between cell-wall properties and the plant environment is poorly understood, and there is little information on the impact of different environmental conditions on cell-wall quality in miscanthus. A previous study showed that drought treatment significantly increases enzymatic saccharification of cellulose in miscanthus, with a concomitant increase in the relative proportion of hemicelluloses (van der Weijde *et al.*, 2017). On the other hand, a different study showed that nitrogen treatments had little impact on biomass composition traits (Dierking *et al.*, 2016).

While water and nutritional stresses are mostly studied in isolation, they are often experienced in combination, particularly when crops are grown on marginal land. For this reason, and when projected changes in climate are considered, understanding the combined effects of these stresses is important. In this study, three different miscanthus genotypes were exposed to drought and nutrient stresses, both separately and in combination, with the aim of evaluating their impact on plant growth and cell-wall properties. Automated imaging facilities at the National Plant Phenomics Centre (NPPC-Aberystwyth) were used for dynamic phenotyping to identify plant responses to separate and combinatorial stresses. Recent studies have demonstrated differences in cell-wall properties and recalcitrance to deconstruction between miscanthus leaves and stems (da Costa *et al*., 2014, 2017). Accordingly, harvested leaf and stem samples were separately subjected to saccharification assays and cell-wall composition analyses. Our results provide insights for future studies to improve miscanthus resilience on marginal land and in future climate scenarios, regarding both yield and cell-wall quality traits.

## MATERIALS AND METHODS

### Experimental design

**Fig. 1.**
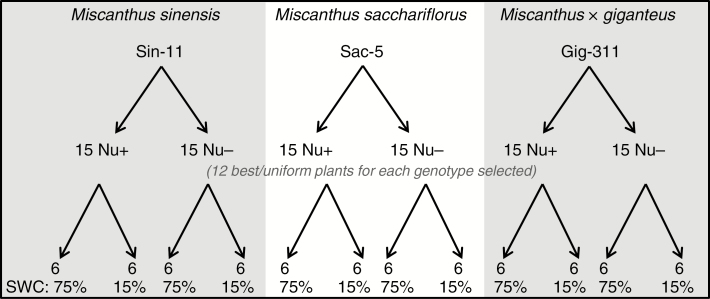
Overview of the different experimental treatments. For each of the three miscanthus genotypes, 30 plants were grown from rhizome material: 15 in Nu+ and 15 in Nu− compost. For six plants per genotype, watering was stopped 26 d after planting until SWC reached 15 %. The remaining six selected plants continued to receive water at 75 % SWC. After 30 d of water treatments, above-ground biomass was harvested for further analyses.

A replicated three-factor experiment was conducted under controlled environment conditions to compare three miscanthus genotypes (one each of *M. sinensis*, *M. sacchariflorus* and *M. × giganteus*), two soil fertility treatments (high and low), two irrigation treatments (well-watered and drought) and their combinations (Fig. 1). Plants were initially established under well-watered conditions for 26 d (16 d in the preparation room followed by 10 d in the gravimetric watering facility of NPPC at Aberystwyth University, followed by 30 d of differential water treatments.

### Plant material and phenotyping

The miscanthus genotypes studied were *Miscanthus sinensis* Sin-11 (= EMI-11, diploid; Clifton-Brown *et al*., 2001), *Miscanthus sacchariflorus* Sac-5 (= EMI-5, tetraploid) and *Miscanthus × giganteus* Gig-311 (triploid), a naturally occurring hybrid of diploid *M. sinensis* and tetraploid *M. sacchariflorus* that is grown commercially in Europe. The three genotypes of miscanthus studied here originated from Japan but were included in previous studies conducted in Europe (Clifton-Brown *et al*., 2001; Purdy *et al.*, 2013). Rhizomes from these miscanthus genotypes were collected in February 2015 from field-grown plants in Aberystwyth, UK. After storage in containers filled with sand at 4 °C, rhizomes were planted in April 2015 in individual square pots (15 cm × 15 cm, 20 cm deep). For each genotype, 15 matching pairs of rhizomes of similar size were prepared. One of each pair was placed in Levington F2 compost [nutrient-rich (Nu+): pH 5.3–6.0, N144:P73:K239 ppm] and the other in 50 % Levington F1 [nutrient-low (Nu−): pH 5.3–6.0, N96:P49:K159 ppm] mixed with 50 % grit sand with no measurable nutrient. Pots were filled with uniform weights of each of the composts and samples were taken for dry matter and field capacity measurements, and soil water content was calculated as the difference between these two values. Target watering weight was calculated based upon a percentage of the water content. The potted rhizome material was placed in one of the preparation rooms of the NPPC at Aberystwyth University.

Sixteen days after planting, the plants were transferred to a gravimetric watering facility in a randomized block design. Each plant was placed on a watering station, which consisted of an electronic balance (Kern FCB12K1) linked through a USB hub to a Rasp Pi computer and a data server. The weight of the plant was monitored at 5-min intervals and water was added twice daily to return the plant to its original target weight. All plants were initially watered to 75 % soil water content (SWC) for 10 d. At this stage, the 12 most uniform plants, out of the original 15 plants available for each genotype and compost level, were selected for further analysis.

Twenty-six days after planting, six plants per genotype and nutrient level were subjected to drought treatment (reaching 15 % SWC) while the remaining six plants continued to be watered to 75 % SWC (see Fig. 1 for overview). The custom-built gravimetric system (Supplementary Data Fig. S1) allowed automated weighing and watering of the plants. Samples of the compost were soaked to determine field capacity or dried to determine dry matter content. Watering treatments lasted for a total of 30 d, after which all above-ground material was harvested. Plants were imaged at weekly intervals using a LemnaTec Scanalyzer 3D (LemnaTec, Aachen, Germany). Three high-resolution visual spectrum images (2056 × 2454 pixels) were taken of every plant: two side views differing from a 90° rotation and a top view.

### Feature extraction

Feature extraction from the RGB colour images was essentially as previously described (Fisher *et al.*, 2016). Images were processed to segment the plant from the background and to extract plant height and side-view projection areas together with colour information. A pixel was classified as yellow if the red part of the RGB value was strictly greater than the green value plus 10.

### Physiological measurements

Image-based phenotyping was complemented by weekly physiological measurements (see Ings *et al.*, 2013 for more details) taken from equivalent leaves and from the tallest stem (at the beginning of the experiment) where multiple stems were present.

Stomatal conductance was measured on the youngest leaf with a fully expanded ligule (leaf 0) using an AP4 porometer (Delta-T Devices, Cambridge, UK). Chlorophyll fluorescence was measured on leaf 0 with a Handy PEA continuous excitation chlorophyll fluorimeter (Hansatech Instruments, King’s Lynn, UK). Chlorophyll content was measured using a SPAD-502 m (Konica Minolta Optics). Plant water content was evaluated from total above-ground biomass measurements taken at the end of the experiment. Fresh weight (FW) was recorded at harvest and dry weight (DW) was the constant weight achieved after drying in a 60 °C oven up to constant weight. The FW and DW figures were then used to calculate above-ground plant water content (PWC) on an FW basis (Fisher *et al.*, 2016).

### Preparation of cell-wall material

After the 30 d of watering treatments (56 d after planting), alcohol-insoluble residue (AIR) was prepared from leaf (blade and sheet) and stem tissue for four out of the six biological replicates for each genotype and treatment. Leaf and stem samples were milled, passed through a perforated plate screen containing 2-mm diameter holes, and 100 mg was weighed into 2-mL tubes. The removal of starch was based on the procedure described by Fry (1988) and previously used to dissolve starch in a variety of grasses, including miscanthus (Gomez *et al.*, 2008; De Souza *et al.*, 2015). Samples were washed with 96 % ethanol, air-dried and incubated overnight with 90 % aqueous DMSO (20:1 v/v ratio) in a shaking incubator at room temperature. Samples were collected by centrifugation, washed three times with 96 % ethanol and dried.

### Saccharification

The cell-wall AIR samples were used for sacchariﬁcation assays, with four technical replicates for each sample, using an automatic platform as previously described by Gomez *et al.* (2010). The biomass was subjected to a mild pretreatment (water at 94 °C for 30 min) and subsequently subjected to sacchariﬁcation using a 4:1 mixture of Celluclast and Novozyme 188 with an enzyme loading of 9 filter paper units (FPU)/g. Sacchariﬁcation was measured after 8 h by colorimetric detection of reducing sugar equivalents as described by Whitehead *et al.* (2012).

### Matrix monosaccharide composition

Cell-wall samples were hydrolysed with 2 m trifluoroacetic acid (TFA) for 1 h at 100 °C. The acid was evaporated under vacuum and the monosaccharides were resuspended in 2 mL of ultra-purified water. Monosaccharide profiles were analysed by high-performance anion exchange chromatography with pulsed amperometric detection (HPAEC-PAD) on a CarboPac SA10 column (DX-500 system, Dionex) using a mixture of 99.2 % water and 0.8 % (v/v) 150 mm NaOH as eluent (1 mL min^−1^). The monosaccharides were detected with a post-column addition of 500 mm NaOH (1 mL min^−1^).

### Crystalline cellulose content

The residue following matrix monosaccharide composition determinations was used for cellulose determination, using a method adapted from Foster *et al.* (2010). Briefly, 72 % sulphuric acid was added to the pellet samples, followed by incubation at 50 °C, initially for 30 min and then for an additional 15 min following vortex mixing. Samples were diluted to 4 % H_2_SO_4_, neutralized with CaCO_3_ and centrifuged at 2500 *g* for 5 min. One hundred microlitres of the supernatant was mixed with 900 µL of 0.005 m H_2_SO_4_ containing 0.005 m crotonic acid as an internal standard. The mixtures were filtered through 0.45-µm syringe filters (Millipore Corporation, Billerica, MA, USA) and 25 µL was analysed on a high-performance liquid chromatography (HPLC) system fitted with a refractive-index detector (Jasco, Great Dunmow, UK) equipped with a Rezex ROA-organic acid H+ column (Phenomenex, Torrance, CA, USA) at 35 °C, with a 0.005 m H_2_SO_4_ mobile phase flowing at 0.6 mL min^−1^ for 16 min. The concentration of hydrolysed cellulose in the supernatant was determined using a concentration gradient of a glucose standard.

### Lignin content

Lignin content was determined using the acetyl bromide soluble lignin assay (ABSL) following the general procedures described by Fukushima and Hatfield (2004) with modifications as described by da Costa *et al.* (2014). Lignin content was determined in triplicate for all of the 75 % SWC miscanthus samples (3 genotypes × 2 tissues × 2 nutrient levels × 3 technical replicates × 4 plant replicates).

### Statistical analysis

All calculations for descriptive statistics, analyses of variance and Tukey’s range tests were performed using the statistical software Statistica (v. 8.0; StatSoft, Tulsa, OK, USA) at a 5 % significance level (*α* = 0.05). Effect sizes were calculated as *η*^2^ statistics (Cohen, 1973; Levine and Hullett, 2002): *η*^2^ = SS_effect_/SS_total_, where SS is the sum of squares.

## RESULTS

### Effect of nutrient and drought stress on miscanthus phenotypes

**Fig. 2.**
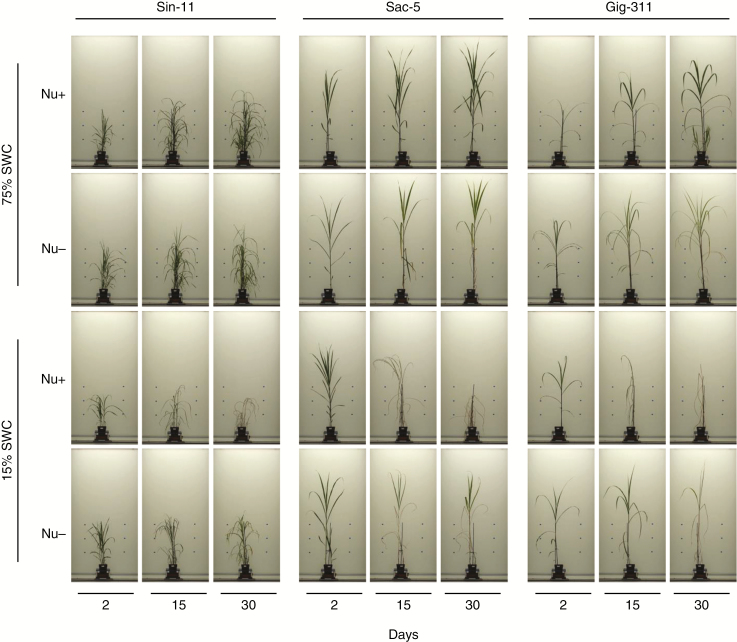
Representative side-image photographs of the three miscanthus genotypes grown in either Nu+ or Nu− compost and exposed to either well-watered (75 % SWC) or drought (15 % SWC) conditions. Photographs are from 2, 15 and 30 d after initiation of the drought treatment. The same biological replicate is shown for each genotype per treatment.

Representative photographs of the three miscanthus genotypes during the course of the experiment when exposed to different treatments are shown in Fig. 2. Under all conditions assayed, the *M. sacchariflorus* genotype (Sac-5) and the *M. × giganteus* genotype (Gig-311) presented similar phenotypic responses. The plant architectures of these two genotypes were different from that of *M. sinensis* (Sin-11). Plants grown in Nu+ compost were more affected by drought than plants grown in Nu− compost. For all three genotypes, plants started to show severe wilting 15 d after initiation of the drought treatment. Plants in Nu− compost appeared to cope remarkably well with drought, in particular Sin-11 (Fig. 2), even though the difference in texture properties between Nu− and Nu+ compost indicated that 15 % SWC was achieved faster for Nu− compared with Nu+ (~3 d versus ~9–12 d; Supplementary Data Fig. S2). Phenotypic features (height, area and colour) extracted from the side-view RGB plant images taken weekly corroborated the visual descriptions.

### Nutrient stress under well-watered conditions

**Fig. 3.**
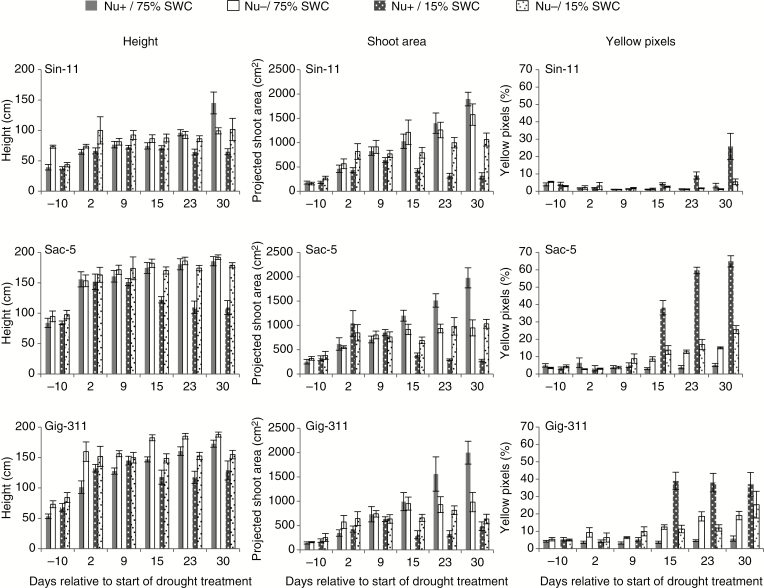
Height, shoot areas and percentage yellow pixels for each of the three miscanthus genotypes for each treatment (Nu+/75 % SWC; Nu−/75 % SWC; Nu+/15 % SWC; Nu−/15 % SWC) as extracted from RGB side images of the plants from 10 d before the start of drought treatment to 30 d after the start of the treatment. For each plant, images used for feature extraction were taken from two side-view angles with an interval of 90°. Data represent averages from six biological replicates for each genotype and treatment. Error bars indicate standard error.

Within genotypes, there was little overall difference in height between plants growing in Nu+ and Nu− compost under well-watered conditions, except for Sin-11 after 30 d. There was a trend for increased height in Nu− compared with Nu+ in Gig-311 under well-watered conditions (Fig. 3). However, digital shoot areas for well-watered Sac-5 and Gig-311 plants showed that above-ground growth ceased from 15 d onwards under Nu− conditions, exhibiting less than half the shoot area at day 30 when compared with plants grown in Nu+ compost (Fig. 3). This nutrient effect was less pronounced in Sin-11, with the average Nu− shoot area reaching 83 % of that for Nu+ at the end of the experiment under well-watered conditions. Despite its different phenotypic characteristics, the digital shoot area for Sin-11 was around 95 % of that of Sac-5 and Gig-311 under well-watered Nu+ conditions and >60 % higher compared with the other two genotypes under well-watered Nu− conditions.

These findings were corroborated by final shoot FW measurements, showing similar values for the three genotypes for Nu+/75 % SWC and a higher value for Nu−/75 % SWC Sin-11 when compared with Sac-5 and Gig-311 (18 and 24 % higher, respectively; Supplementary Data Fig. S3). Furthermore, weight measurements indicated a higher contribution of leaves to the overall biomass accumulation in Sin-11. For this genotype, the leaf-to-stem ratio was 2.9 and 2.5 for FW and DW, respectively under Nu+ conditions. For Sac-5, the ratio was 0.9 for both FW and DW, while for Gig-311 the ratio was 1.2 and 1.0 for FW and DW, respectively (see also Supplementary Data Fig. S3). Interestingly, when compared with Nu+, the leaf-to-stem ratios for FW and DW increased for Sin-11 under Nu− conditions (from 2.9 to 3.3 and from 2.5 to 3.5, respectively), whereas they decreased for both Sac-5 (FW ratio from 0.9 to 0.6, DW ratio from 0.9 to 0.4) and Gig-311 (FW ratio from 1.2 to 0.6 and DW ratio from 1.0 to 0.5). ANOVA results of the weight measurements are included in Supplementary Data Tables S1 and S2).

The relative yellow pixel content, a feature associated with leaf senescence and stress response symptoms, was ~3-fold higher under Nu− conditions compared with Nu+ from day 15 onwards for Sac-5 and Gig-311, reaching 15 % for Sac-5 and almost 20 % for Gig-311 at the end of the experiment. Little yellowing occurred in Sin-11 when well watered, irrespective of nutrient condition, the yellow pixel contribution remaining <5 % (Fig. 3). Under Nu− conditions, photosynthetic measurements taken on the youngest leaf with a fully expanded ligule (leaf 0) had 25–35 % lower chlorophyll fluorescence for the three genotypes than those from Nu+ plants towards the end of the experiment (Supplementary Data Fig. S4A). No significant differences were observed in stomatal conductance or chlorophyll fluorescence between plants grown in Nu+ and Nu− compost under well-watered conditions (Supplementary Data Fig. S4B, C).

Together, these phenotypic analyses indicate that, when exposed to nutrient-poor compost under well-watered conditions, Sin-11 sustained better overall growth characteristics compared with Sac-5 and Gig-311.

### Nutrient stress under conditions of drought

Height and shoot area under drought (15 % SWC) were significantly lower for plants grown in Nu+ compost (compared with those plants grown in Nu− compost) (Fig. 3). These findings were consistent with severely senesced plants observed by days 15 and 30 (Fig. 2) and with high yellow pixel content, particularly for Sac-5 (day 15, 38 %; day 30, 65 %) and Gig-311 (day 15, 39 %; day 30, 37 %). Sin-11 exhibited lower yellow pixel content under these conditions (day 15, 4 %; day 30, 26 %). Sin-11 and Sac-5 achieved similar heights in 75 % SWC or 15 % SWC in Nu− conditions. The digital shoot area for these two genotypes was similar under Nu−/15 % SWC conditions (averaging 1064 and 1033 cm^2^, respectively). The shoot area of Sin-11 was 30 % smaller compared with the area at Nu−/75 % SWC, while the area for Sac-5 remained more or less constant irrespective of watering regime under Nu− conditions. Digital shoot area for Gig-311 at Nu−/15 % SWC was almost 40 % lower compared with Sin-11 and Sac-5, although this did not translate to similar weight reduction (Supplementary Data Fig. S3). Substantial yellowing could be observed in Sac-5 and Gig-311 plants exposed to Nu−/15 % SWC, both reaching 25 %, while this was only 5.5 % for Sin-11 (Fig. 3).

Photosynthetic measurements showed no major differences between Nu− plants at either 75 % SWC or 15 % SWC (Supplementary Data Fig. S4). However, under Nu+ conditions, drought treatment increased stomatal resistance (indicating decreased stomatal size and conductance) 15 d after the start of the watering treatments (Supplementary Data Fig. S4). Severely senesced leaf material limited the extent to which photosynthetic measurements could be reliably recorded towards the end of the experiment. Overall, under these experimental conditions, phenotypic measurements showed that Sac-5 and particularly Sin-11 coped better than Gig-311 with drought stress when grown in Nu- compost.

### Effect of drought and nutrient stress on biomass saccharification

**Fig. 4.**
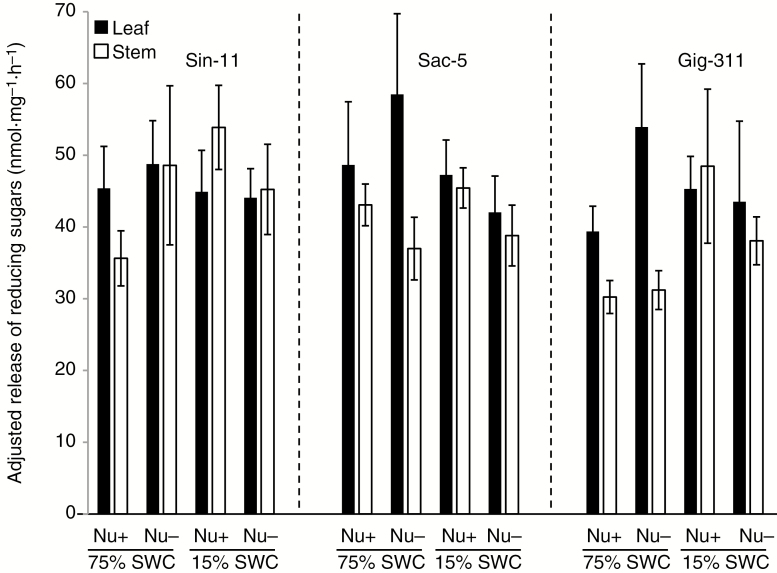
Saccharification of cell-wall material from harvested miscanthus leaf and stem samples after the experimental treatments. The cell-wall biomass was subjected to a mild pretreatment (water at 94 °C for 30 min) before enzymatic saccharification. Sugar release was measured by colorimetric detection of reducing sugar equivalents. Data represent averages from four biological replicates for each genotype and treatment, with four technical replicates for each sample. Outliers were excluded by the *z*-score calculation method (excluded *z*-score >4). Error bars indicate standard deviation. The saccharification data are tabulated in Supplementary Data Table S4.

To investigate whether the different evaluated stress conditions modify the saccharification potential of the biomass produced by the three miscanthus genotypes, enzymatic saccharification assays were performed on leaf and stem biomass samples harvested at the end of the phenomics experiment. Both in leaves and in stems, the variation in nutrition and SWC levels had significant effects on the saccharification yields of the plants (*P* < 0.05; Supplementary Data Table S3). Under Nu+/75 % SWC conditions, sugar release was highest for Sac-5, followed by Sin-11 and Gig-311 for leaf and stem biomass (Fig. 4 and Supplementary Data Table S4), with sugar release between Sac-5 and Gig-311 being significantly different (*P* < 0.05; Supplementary Data Table S5). As a trend, under these conditions sugar release was typically higher from leaves compared with stems for all three genotypes (28, 13 and 30 % higher for leaves from Sin-11, Sac-5 and Gig-311, respectively). However, a significant difference (*P* < 0.05) between leaves and stems from plants under Nu+/75 % SWC was only detected for genotype Sin-11. Sugar release from leaf samples taken from Nu+ plants was not significantly affected by SWC. Likewise, at 15 % SWC, there were no significant differences in sugar release from Nu+ and Nu− leaf samples. However, under Nu− conditions, exposure to 15 % SWC significantly reduced sugar release from leaves for Sac-5 and Gig-311 (reduction of 28 and 19 %, respectively; *P* < 0.05; see also Supplementary Data Table S5) when compared with 75 % SWC. Interestingly, leaves from these two genotypes also showed significantly higher sugar release for Nu− compared with Nu+ at 75 % SWC (20 and 37 %, respectively; *P* < 0.05; Fig. 4 and Supplementary Data Tables S4 and S5). In contrast to leaf samples, SWC had no significant effect on sugar release from Nu− stem samples. At 15 % SWC, Nu+ Sin-11 and Gig-311 stem samples exhibited significantly higher sugar release (both >50 %; *P* < 0.05) than Nu+/75 % SWC samples (Fig. 4, Supplementary data Tables S4 and S5). For 15 % SWC, sugar release was significantly higher for Sin-11 and Gig-311 stems in Nu+ compared with Nu− (19 and 36 % higher, respectively; *P* < 0.05). In contrast, 75 % SWC resulted in significantly higher sugar release for Sin-11 Nu− stem samples compared with corresponding Nu+ samples (36 % higher; *P* < 0.05). Across the genotypes, water stress had little impact on saccharification of leaves under Nu+ conditions and of stems under Nu− conditions. For Sac-5 and Gig-311, sugar release was sensitive to SWC under Nu− conditions and to nutrient availability under well-watered conditions. Sugar release from Sin-11 stems was more affected by water and nutrient availability compared with leaves.

Overall, the data suggest that a combination of well-watered conditions and low nutrient availability can lead to the highest sugar release from leaves. For stems, the highest sugar release was generally associated with the combination of drought and Nu+ conditions. The results indicate that exposure to different environmental conditions, in this case water and nutrient levels, can have different consequences for the sugar release characteristics of young leaves and stems.

### Do different levels of nutrient availability affect cell-wall composition?

Since the sugar release for all of the three genotypes was sensitive to nutrient levels under well-watered 75 % SWC conditions, which also yielded significantly higher levels of above-ground biomass when compared with 15 % SWC (Fig. 3 and Supplementary data Fig. S3), we analysed cell-wall composition in the 75 % SWC samples for comparison with the saccharification data. Table 1 shows the percentage of the monosaccharides in the non-crystalline fraction of cell walls from the three genotypes grown at 75 % SWC. Overall, there was little difference in matrix monosaccharides between the Nu+ and Nu− samples of a given genotype. The only statistically significant difference (*P* < 0.05) was observed for glucose, the second most abundant matrix monosaccharide, being >4 times higher in Sac-5 Nu− stem samples compared with Sac-5 Nu+ stem samples (Table 1 and Supplementary Data Table S7b). Although matrix glucose was 3 times higher in Sac-5 Nu− leaves compared with those of Sac-5 Nu+ and more than 2 times higher in Nu− Sin-11 stems compared with Nu+ stems, both of these results were not statistically significant. It is important to note that the TFA hydrolysis protocol releases glucose preferentially from mixed-linkage β-glucan and xyloglucan rather than cellulose. Xylose levels were highest in stems of Sin-11, while arabinose and galactose (the latter predominantly associated with pectin) were in all cases highest in Sin-11 samples, and for all genotypes higher in leaf compared with stem samples.

**Table 1.** Matrix monosaccharide content (expressed as percentage of total DW biomass)

	Leaf		Stem	
Sugar, genotype	Nutrient-poor	Nutrient-rich	Nutrient-poor	Nutrient-rich
Fucose				
Sin-11	0.04 ± ≤0.01	0.04 ± ≤0.01	0.02 ± ≤0.01	0.02 ± ≤0.01
Sac-5	0.04 ± 0.01	0.04 ± ≤0.01	0.01 ± ≤0.01	0.01 ± ≤0.01
Gig-311	0.04 ± 0.01	0.04 ± ≤0.01	0.01 ± 0.01	0.01 ± ≤0.01
Arabinose				
Sin-11	4.27 ± 0.23	4.12 ± 0.36	3.04 ± 0.52	2.83 ± 0.33
Sac-5	3.73 ± 0.25	3.47 ± 0.30	1.53 ± 0.18	1.77 ± 0.13
Gig-311	3.76 ± 0.23	3.92 ± 0.09	2.09 ± 0.92	1.62 ± 0.14
Galactose				
Sin-11	1.11 ± 0.06	1.04 ± 0.07	0.61 ± 0.11	0.44 ± 0.06
Sac-5	0.84 ± 0.07	0.79 ± 0.08	0.19 ± 0.03	0.25 ± 0.05
Gig-311	0.88 ± 0.07	0.93 ± 0.06	0.42 ± 0.27	0.30 ± 0.05
Rhamnose				
Sin-11	0.04 ± 0.01	0.03 ± 0.02	0.02 ± 0.01	0.03 ± 0.01
Sac-5	0.03 ± ≤0.01	0.04 ± ≤0.01	≤0.01 ± ≤0.01	0.02 ± 0.01
Gig-311	0.03 ± ≤0.01	0.04 ± 0.01	0.02 ± 0.02	0.01 ± 0.01
Glucose				
Sin-11	7.84 ± 3.62	5.56 ± 3.74	12.95 ± 6.67	4.54 ± 2.29
Sac-5	5.67 ± 2.74	1.86 ± 1.20	11.87 ± 2.39	2.62 ± 0.92
Gig-311	3.12 ± 1.39	3.12 ± 1.29	7.81 ± 4.17	7.20 ± 4.14
Xylose				
Sin-11	15.76 ± 1.94	15.52 ± 1.32	17.37 ± 1.11	18.10 ± 1.47
Sac-5	16.08 ± 0.68	15.76 ± 1.33	13.27 ± 1.03	15.30 ± 0.58
Gig-311	15.41 ± 0.74	16.08 ± 0.27	13.20 ± 1.66	12.92 ± 0.83
Mannose				
Sin-11	0.09 ± 0.01	0.10 ± 0.02	0.05 ± ≤0.01	0.06 ± 0.01
Sac-5	0.08 ± 0.02	0.11 ± 0.01	0.02 ± ≤0.01	0.03 ± 0.01
Gig-311	0.10 ± 0.04	0.09 ± 0.01	0.04 ± 0.03	0.03 ± ≤0.01

Values are mean ± standard deviation of biological and technical replicates (minimum for each value, *n* = 4; total samples for each monosaccharide, *n* = 60).

Leaf and stem samples used for this assay were collected at the end of the experiment from the three miscanthus genotypes grown at 75 % SWC.

Supplementary Data Table S6 shows the ANOVA results for the matrix monosaccharides.

No significant differences in crystalline cellulose were observed between Nu+ and Nu− samples of the three genotypes (Table 2 and Supplementary Data Table S9). For leaves, crystalline cellulose content was lowest in Sin-11 and highest in Gig-311, while for stem samples it was lowest for Sac-5 Nu− and highest for Sac-5 Nu+.

**Table 2.** Crystalline cellulose content measured as percentage of cell-wall biomass DW

	Leaf		Stem	
Genotype	Nutrient-poor	Nutrient-rich	Nutrient-poor	Nutrient-rich
Sin-11	28.74 ± 4.66	25.63 ± 1.27	33.66 ± 5.41	29.86 ± 1.85
Sac-5	31.04 ± 3.59	28.31 ± 5.78	27.44 ± 2.06	34.23 ± 5.37
Gig-311	32.32 ± 5.34	34.87 ± 2.89	33.65 ± 6.35	33.68 ± 6.19

Values are mean ± standard deviation of biological and technical replicates (minimum for each value, *n* = 8; total samples, *n* = 96).

Leaf and stem samples used for this assay were collected at the end of the experiment from the three miscanthus genotypes grown at 75 % SWC.

Outliers were excluded via the *z*-score calculation method (excluded *z*-score >4).

Supplementary Data Table S8 shows the ANOVA results for the cellulose content.

Overall, lignin content was significantly lower in leaves compared with stems (*P* < 0.05; Supplementary Data Table S10). Stem samples all exhibited similar lignin content, except for Sin-11 Nu−, which showed typically lower values when compared with the stem samples of the other genotypes. Within the same genotype, lignin content was only significantly different between Sac-5 Nu+ and Nu− stem samples, with a significantly higher amount of acetyl bromide-soluble lignin in Nu− (*P* < 0.05; Table 3 and Supplementary Data Table S11).

**Table 3.** Acetyl bromide-soluble lignin content (ABSL) as percentage of cell-wall biomass DW

	Leaf		Stem	
Genotype	Nutrient-poor	Nutrient-rich	Nutrient-poor	Nutrient-rich
Sin-11	15.08 ± 1.49	14.60 ± 0.87	15.76 ± 1.80	16.65 ± 1.47
Gig-311	16.24 ± 0.87	15.78 ± 4.35	17.00 ± 1.40	16.78 ± 1.54
Sac-5	15.40 ± 0.77	16.14 ± 1.17	18.13 ± 1.45	16.16 ± 0.87

Values are mean ± standard deviation of biological and technical replicates (for each value, *n* = 3; total samples, *n* = 144).

Leaf and stem samples used for this assay were collected at the end of the experiment from the three miscanthus genotypes grown at 75 % SWC.

## DISCUSSION

The cultivation of perennial biomass crops on abandoned agricultural land, degraded land, reclaimed land and wasteland can reduce the competition between food and biomass crops for land use with the potential to support biodiversity and soil carbon sequestration (Carlsson *et al.*, 2017). Perennial C4 grasses from the genus *Miscanthus* combine high biomass production potential with adaptability to different environmental conditions and low requirements for agricultural inputs (Lewandowski *et al.*, 2000; Clifton-Brown *et al.*, 2017). Therefore, miscanthus is particularly well suited for biomass production on marginal sites.

However, the relationship between the levels of water and nutrients, which are two key limiting resources for the cultivation of miscanthus on marginal land (FAO, 2011), and their possible impacts on biomass quality are poorly understood. Here we have determined the individual and combined effects of drought and nutrient levels on the growth and development of three miscanthus genotypes during the early establishment phase. We show that the experimental environmental stresses influence sugar release and composition of cell-wall-derived lignocellulose, a key biomass quality parameter.

### Differential growth responses of miscanthus to imposed environmental conditions

Although a number of previous studies have focussed on the effects of water deficit for miscanthus growth and development (e.g. Clifton-Brown *et al.*, 2002; Ings *et al.*, 2013; Malinowska *et al.*, 2017; van der Weijde *et al.*, 2017), plants in the field are frequently exposed to multiple environmental stresses, since nutrient and water requirements are closely related. Drought stress conditions generally cause a decrease in the nutrient diffusion rate from the soil matrix to the absorbing root surface, reducing the availability, uptake, translocation and metabolism of nutrients (Farooq *et al.*, 2009). Drought also reduces plant transpiration, resulting in reduced water absorption in the roots (Hu and Schmidhalter, 2005; Farooq *et al.*, 2009; da Silva *et al.*, 2011). However, the exact relationship between the impact of drought on the uptake of nutrients and the subsequent effects on plant physiology remains poorly understood (Ahanger *et al.*, 2016).

In this study, we initially assessed the differences in miscanthus growth response based on genotype, irrigation treatment (15 versus 75 % SWC) and nutrient levels (Nu+ and Nu−). Under relative nutrient-poor (Nu−) conditions, the levels of nitrogen, phosphorus and potassium macronutrients (N-P-K) in the compost were reduced to about one-third of the N-P-K levels in Nu+ compost. Using the imaging facilities of the UK National Plant Phenomics Centre (NPPC-Aberystwyth), we observed a strong correlation between biomass obtained from digital image analysis and destructive biomass analysis at the endpoint of the irrigation treatments, with *R*^2^ values of 0.91 and 0.79 for FW and DW, respectively. These values correspond well with the *R*^2^ values of 0.92 and 0.84 obtained in a previous study for FW and DW measures, respectively, when 47 miscanthus genotypes were exposed to water stress and imaged using the NPPC facilities (Malinowska *et al.*, 2017).

The percentages relative to the respective Nu+/75 % SWC measures for shoot area, and for both FW and DW measurements at the end of the experiments for all the different treatments and genotypes are presented in Table 4. The experiments showed that *M. sinensis* Sin-11 performed better than *M. sacchariflorus* Sac-5 and *M. × giganteus* Gig-311 under well-watered but nutrient-poor conditions (Nu−/75 % SWC). The growth of all three miscanthus genotypes was most severely compromised when exposed to drought using Nu+ compost, as characterized by highly reduced shoot area, lower above-ground biomass accumulation and significant wilting. Miscanthus genotypes were resilient to drought stress under nutrient-poor conditions, and in particular Sin-11 and Sac-5 showed a robust response under Nu−/15 % SWC conditions. Both these genotypes achieved projected shoot area and DW measures of >50 % as compared with Nu+/75 % SWC conditions (Table 4). Both these genotypes also showed moderate (Sac-5) or little (Sin-11) signs of wilting and/or chlorosis. A previous study also showed *M. sinensis* to be less sensitive to water stress when compared with *M. × giganteus* and *M. sacchariflorus*, with little signs of leaf senescence, and was referred to as a stay-green phenotype (Clifton-Brown *et al.*, 2002).

**Table 4.** Biomass measurements expressed as relative percentages

	Shoot area			
Genotype	Nu+/75 % SWC	Nu−/75 % SWC	Nu+/15 % SWC	Nu−/15 % SWC
Sin-11	100 (95)	83 (79)	17 (16)	56 (53)
Sac-5	100 (99)	48 (47)	14 (14)	52 (52)
Gig-311	100 (100)	49 (49)	24 (24)	32 (32)
	Fresh weight			
	Nu+/75 % SWC	Nu−/75 % SWC	Nu+/15 % SWC	Nu−/15 % SWC
Sin-11	100 (109)	73 (79)	11 (12)	30 (33)
Sac-5	100 (101)	66 (67)	23 (23)	51 (51)
Gig-311	100 (100)	63 (63)	23 (23)	40 (40)
	Dry weight			
	Nu+/75 % SWC	Nu−/75 % SWC	Nu+/15 % SWC	Nu−/15 % SWC
Sin-11	100 (91)	95 (86)	27 (24)	52 (48)
Sac-5	100 (97)	88 (86)	29 (28)	59 (57)
Gig-311	100 (100)	84 (84)	25 (25)	47 (47)

Values for Sin-11, Sac-5, and Gig-311 at Nu+/75 % SWC were set at 100 %.

Biomass measures for the various treatments for a given genotype are relative to those at Nu+/75 % SWC.

Relative percentages for the biomass measurements in this table are derived from absolute measurements (see Supplementary data Fig. S3) taken from the miscanthus genotypes at the end of the experimental treatments.

Percentage values in brackets are all relative to those of Gig-311 at Nu+/75 % SWC.

Under drought stress, the addition of plant nutrients is generally considered a favoured strategy to enhance water use efficiency and productivity in crop plants (Waraich *et al.*, 2011), unless the drought stress is too severe (Hu and Schmidhalter, 2005). The data obtained in this study show that drought stress in combination with high levels of N-P-K (Nu+) lead to a dramatic effect on miscanthus growth, with plants becoming severely wilted. These results contrast with findings in maize seedlings, where N-P-K fertilization increased the growth rate under conditions of drought stress (Studer *et al.*, 2007). Several studies have shown that nitrogen treatment can partly alleviate water stress-associated damage in plants (Dubey *et al.*, 2001; da Silva *et al.*, 2011) and a close relationship between potassium nutritional status and plant drought resistance has been demonstrated (Wang *et al.*, 2013). Water-stressed maize plants showed improved adaptation to water deficits at higher potassium levels (Premachandra *et al.*, 1991) and exogenous application of potassium enhanced drought tolerance of wheat and barley (Samar Raza *et al.*, 2013; Fayez and Bazaid, 2014). Although some studies have shown that drought tolerance and water use efficiency can be improved by increased phosphorus (Ahanger *et al.*, 2016), the involvement of phosphorus in alleviating drought is less clear.

The combination of water stress and Nu+ compost resulted in a severe effect on miscanthus during early establishment in the pot experiment. Generally, minimal N-P-K fertilizer is recommended during the establishment phase of *M. × giganteus* (Cadoux *et al.*, 2014; Haines *et al.*, 2015). The UK Department for Environment, Food & Rural Affairs (DEFRA) has recommended values of 10–15 mg L^−1^ phosphorus, 61–120 mg L^−1^ potassium and a minimum of 150 kg ha^−1^ nitrogen during the miscanthus establishment phase (DEFRA, 2001). When compared with these recommendations, the level of phosphorus was ~2-fold higher in our Nu− compost (24.5 mg L^−1^) and almost 5-fold higher in Nu+ (73 mg L^−1^) compost. Potassium in our Nu− compost (79.5 mg L^−1^) was within the recommended range, while it was 2-fold higher in Nu+ compost. The level of nitrogen in the two composts tested equates to ~220 kg ha^−1^ for Nu+ and 70 kg ha^−1^ for Nu−, respectively, equivalent to ~147 and 47 % of the recommended minimum level. The interaction between water stress and nutrient levels and the effect on plant growth and development depend on plant species, developmental stage, drought stress levels, nutrient levels and soil characteristics. The higher levels of fertilizer salts in Nu+ compost are associated with higher conductivity of the compost (Nu+, 210–290 μS; Nu−, 155–215 μS before addition of 50 % grit sand). Although commercial composts generally have a reduced risk of ‘fertilizer burn’, the combination of high N-P-K and low water levels may have resulted in a high osmotic concentration of salts in the compost that aggravated the drought-induced osmotic stress. However, it has been shown that, when grown on saline soil that induces water shortage due to osmotic stress and accumulation of salt in the plant, some *M. sinensis* and *M. sacchariflorus* genotypes exhibit salinity tolerance while the commercially available *M. × giganteus* genotype is not suited for cultivation on such land (Lewandowski *et al.*, 2016). It is important to emphasize that plants grown in Nu+ compost achieved 15 % SWC 6–8 d later compared with plants grown in Nu− compost, which contained 50 % grit sand. Thus, even though Nu−/15 % SWC plants were exposed to the target drought for a longer period (26–27 d), these plants still performed much better than those exposed to Nu+/15 % SWC, which only experienced target drought for 18–21 d.

In summary, the phenotyping results highlight the fact that miscanthus can establish well under relative nutrient-poor conditions, with Sin-11 being the best-performing genotype at Nu−/75 % SWC of the three genotypes tested and Sin-11 and Sac-5 performing better than Gig-311 at Nu−/15 % SWC. These results for Sin-11 confirm previous studies showing that *M. sinensis* types can tolerate extreme variations in soil and climate conditions (Quinn *et al.*, 2012), reinforcing their status as good candidates for cultivation in stressful environments, including less productive marginal lands.

### Water and nutrient levels affect cell-wall sugar release in a tissue- and genotype-dependent manner

It is important to develop crops that can maintain biomass productivity and quality even when cultivated in conditions that are unsuitable for food crops, such as in poor soils and under water-scarce environments. This becomes even more relevant given the scenario of predicted climate change, where crops must be quickly adapted to challenging environmental conditions. Most of the chemical energy within grass lignocellulosic biomass is located within its cell walls, which comprise a network of cellulose, xylans and lignin polymers that interact to assemble a complex and recalcitrant matrix (McCann and Carpita, 2008). The relative abundances and interactions among the cell-wall polymers dictate biomass recalcitrance, which has been defined as the resistance of plant cell walls to deconstruction into monomeric sugars (Pauly and Keegstra, 2010; DeMartini *et al.*, 2013, De Souza *et al.*, 2014). Recalcitrance can be assessed by measuring saccharification yield, which is the total sugar released by enzymatic treatment of cell-wall polysaccharides. Since the architecture of the cell-wall matrix is affected by abiotic stress conditions, which can result in cell-wall loosening or tightening (Le Gall *et al.*, 2015), this will likely impact on the sugar release from plant biomass.

Indeed, our saccharification results demonstrate that different environmental conditions affect biomass quality in a tissue- and genotype-dependent manner during the early establishment phase of miscanthus. Under nutrient-rich conditions, exposure to drought significantly increased the saccharification yield from stems of Sin-11 and Gig-311 (51 and 60 % increase, respectively), while no significant changes were observed for leaves. These results are in broad agreement with those from a previous study in which 50 miscanthus genotypes exposed to drought showed on average a 20 % higher cellulose conversion efficiency from stems compared with controls, although this study also reported a 7 % increase in the cellulose conversion from leaves (van der Weijde *et al.*, 2017).

The observation that more sugars were released from leaves compared with stems under well-watered nutrient-rich conditions also agrees with previous studies in miscanthus showing that leaves are less recalcitrant compared with stems (Le Ngoc Huyen *et al.*, 2010; da Costa *et al.*, 2014) underpinned by key differences in their cell-wall fine structures and composition (da Costa *et al.*, 2017). Similar differences in recalcitrance between these two organs have also been reported for other grasses, such as wheat (Zhang *et al.*, 2014) and switchgrass (Crowe *et al.*, 2017).

Unexpectedly, low soil macronutrient levels combined with well-watered conditions reduced the recalcitrance of Sac-5 and Gig-311 leaves even further (20 and 37 % increased sugar release compared with Nu+/75 % SWC, respectively), while for Sin-11 these conditions increased the sugar release from stems by 36 %. Potassium fertilization has been suggested to negatively influence biomass combustion quality characteristics in miscanthus by increasing the concentrations of Cl, K, N and ash (Lewandowski and Kicherer, 1997). However, to our knowledge, this is the first report of the effect of different soil nutrient levels on saccharification either in miscanthus or other grasses. The higher sugar release for Sac-5 and Gig-311 leaves under Nu−/75 % SWC conditions was not maintained when plants were exposed to water-limiting conditions (Nu−/15 % SWC). Overall, the saccharification results indicate that, although different environmental conditions may impose a yield penalty on the accumulation of above-ground biomass, under some circumstances such penalties may be offset by enhanced sugar release. This is demonstrated in Supplementary Data Table S12, which presents the sugar release from above-ground biomass based on DW measures, relative to that of Gig-311 under Nu+/75 % SWC conditions. Based on our results Sin-11, when grown under nutrient-poor but well-watered conditions, can achieve 20 % higher total sugar release compared with Gig-311 at Nu+/75 % SWC. Under even harsher Nu−/15 % SWC conditions, both Sin-11 and Sac-5 still release 61 and 66 %, respectively, of the sugars released by Gig-311 at Nu+/75 % SWC. The saccharification data emphasize that exposure to different environmental conditions, in this case water and nutrient levels, can have very different consequences for the sugar release characteristics of leaves and stems. A combination of abundant water and low nutrients can lead to the highest sugar release from leaves, while for stems the highest sugar release is generally associated with the combination of drought and nutrient-rich conditions. It is important to note that biomass yield and saccharification data in this study were obtained from young establishing miscanthus genotypes. Nevertheless, the results indicate (1) that miscanthus has the potential for cultivation as a biomass crop under suboptimal conditions unsuitable for food crops, and (2) the importance of selecting suitable miscanthus genotypes for different environmental conditions.

Although soil water content and the level of soil nutrients both impacted on the levels of sugar release from the cell-wall matrix, subsequent cell-wall analysis focused solely on samples harvested from well-watered miscanthus plants. The rationale for this was that projected sugar yields from the above-ground biomass were significantly higher under these conditions. Besides, although changes in cell-wall composition have been reported for miscanthus exposed to drought stress (van der Weijde *et al.*, 2017), to our knowledge there are no reports on the effect of soil nutrients on sugar release and the compositional cell-wall features that underpin such effects.

**Fig. 5.**
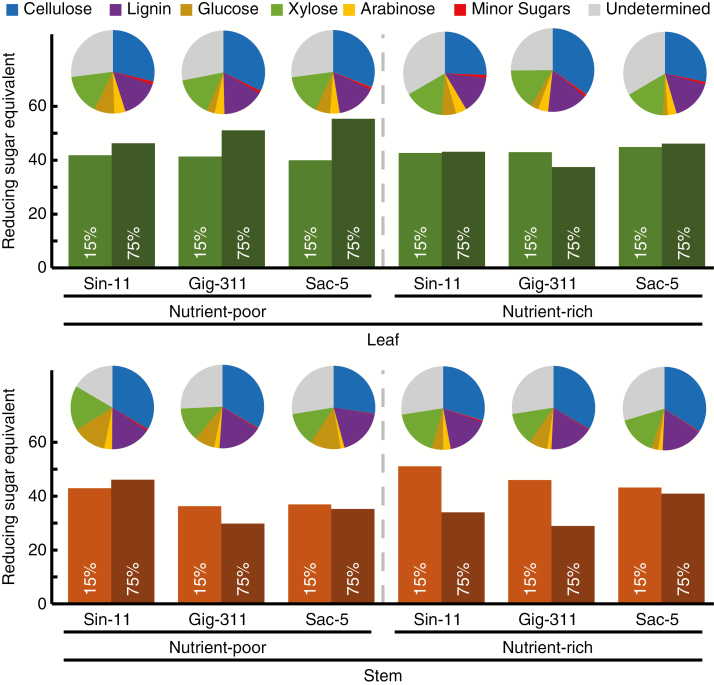
Comparison of the saccharification of lignocellulosic biomass from three miscanthus genotypes. Plants were grown in Nu+ and Nu− conditions and at 15 % or 75 % SWC. Sacchariﬁcation was measured by colorimetric detection of reducing sugar equivalents, and is expressed as nmol of reducing sugars released per mg of dry biomass per hour after 8 h of saccharification. Pie charts indicate the relative (% DW) lignocellulosic biomass composition for leaf and stem of the three miscanthus genotypes grown at 75 % SWC under Nu+ or Nu− conditions. Minor sugars include fucose, galactose and rhamnose. See Tables 1–3 for further details.

Given the large and significant differences in the sugar release data between well-watered Nu+ and Nu− samples, it was noteworthy that we could not detect significant differences in the composition of the matrix polysaccharides, or in cellulose and lignin contents. An overview of the cell-wall composition and sugar release data is shown in Fig. 5. Using detergent fibre analysis, it was previously shown that increased sugar release from miscanthus biomass samples after exposure to drought was accompanied by a reduction in cellulose content and an increase in hemicellulosic polysaccharides, although compositional features did not correlate with drought tolerance (van der Weijde *et al.*, 2017). Results from field trials with *M. × giganteus* also showed that drought significantly affected cell-wall composition, although nitrogen fertilizer levels did not significantly affect composition (Emerson *et al.*, 2014).

Although we could not identify compositional cell-wall differences that correlate with sugar release between well-watered miscanthus genotypes grown in Nu+ and Nu− samples, we believe that a more detailed analysis could reveal the reasons for distinct saccharification levels. This hypothesis is supported by the fact that De Souza *et al.* (2015) demonstrated that not only lignin but also the fine structural features of cell-wall polysaccharides can interfere with saccharification.

The compositional data obtained in this work show that both arabinose and galactose content, as well as the arabinose:xylose (Ara/Xyl) ratio were consistently higher in leaves than in stems (~1.8-, ~2.5- and 1.6- to 1.9-fold higher, respectively) while lignin content was higher in stem material, which is in agreement with previous results for actively growing miscanthus genotypes (da Costa *et al*., 2014, 2017). Considering all samples, it is noteworthy that there was a significant positive correlation (*P* < 0.01) between sugar release and arabinose and galactose content, and with the Ara/Xyl ratio, with Pearson’s correlation coefficients (*r*) of 0.7, 0.6, and 0.6, respectively, and a negative correlation with lignin content (*r* = −0.6; *P* < 0.05) (Supplementary Data Table S13). In grasses, pectins and arabinogalactans are the main Gal-containing cell-wall polysaccharides (Carpita, 1996; O’Neill and York, 2003), while the level of xylan arabinosylation is likely to influence the Ara/Xyl ratio (Rancour *et al.*, 2012). As previously suggested by De Souza *et al.* (2015) and da Costa *et al.* (2017), it is likely that subtle changes in the fine structure of cell-wall polysaccharides, including those of heteroxylans (arabinoxylan and xyloglucan) and pectins, perhaps in association with lignin (including pectin–lignin associations), contribute to the observed differences in saccharification of the cell wall.

The results of this study reinforce the potential of the miscanthus biomass crop for cultivation on marginal land and highlight the importance of the assessment of biomass quality measures in addition to biomass yield determinations. The combination of different irrigation and nutrient treatments had a significant effect on the release of sugars from the cell-wall matrix of leaves and stems, highlighting the importance of genotype–environment interactions. The changes in cell-wall features induced by different abiotic environments that underpin observed sugar release differences have not yet been identified but possibly result from changes in the fine structure of cell-wall constituents. Future studies, using more sophisticated methods for cell-wall analysis and improved measures for nutrient levels and compost texture, could address such changes in more detail. From a commercial point of view, expansion of miscanthus cultivation into marginal agricultural land requires the development of stress-tolerant seed-based hybrids (Clifton-Brown *et al.*, 2017; Hastings *et al.*, 2017). It will therefore be important to evaluate the effect of environmental stresses on the growth and biomass quality measures of such seed-based hybrids, both during the establishment phase as well as in well-established field trials. Miscanthus biomass is normally harvested from mature, fully senesced plants. It will therefore be important to determine to what extent observations made in young miscanthus plants, such as those in this study, are predictive for mature plants. While miscanthus has potential for liquid-based biofuels, future work will also need to address other quality measures associated with the miscanthus biomass-based value-chain products, such as combustion, biogas and other biomaterial requirements (Wagner *et al.*, 2017).

## SUPPLEMENTARY DATA

Supplementary data are available online at https://academic.oup.com/aob and consist of the following. Figure S1: photographs of the gravimetric system used for the watering treatments. Figure S2: average number of days for the three miscanthus genotypes to achieve 15 % soil water content (SWC) after the start of withholding water. Figure S3: weight measures and derived water content (WC) from tissue harvest. Figure S4: photosynthetic productivity measurements. Table S1: ANOVA for the variation in plant fresh weight measurements. Table S2: ANOVA for the variation in plant dry weight measurements. Table S3: ANOVA for saccharification of leaf and stem biomass. Table S4: saccharification assays on cell-wall material from harvested tissue samples. Table S5a: *P*-values of saccharification differences in leaf biomass for each combination of genotype, nutrition level and soil water content. Table S5b: *P*-values of saccharification differences in stem biomass for each combination of genotype, nutrition level and soil water content. Table S6: ANOVA for matrix monosaccharides from leaf and stem separately. Table S7a: *P*-values for significant differences in the leaf content of each matrix monosaccharide between genotypes attributed to nutrition level. Table S7b: *P*-values for significant differences in the stem content of each matrix monosaccharide between genotypes attributed to nutrition level. Table S8: ANOVA for cellulose content of all samples. Table S9: *P*-values for significant differences in cellulose content between genotypes attributed to nutrition level. Table S10: ANOVA for acetyl bromide lignin determinations. Table S11: *P*-values for significant differences in lignin content between genotypes attributed to nutrition level. Table S12: relative sugar release potential (%) based on saccharification results and dry biomass measures. Table S13: Pearson’s correlation coefficient (*r*) between sugar release data and cell-wall content.

mcy155_suppl_aob-18028-s01
