# Supplementary material for: Nutrient and drought stress: implications for phenology and biomass quality in miscanthus
Source: Ann Bot. 2018 Aug 21;124(4):553–66. doi: 10.1093/aob/mcy155 (PMC6821376; doi:10.1093/aob/mcy155)
Supplement: mcy155_suppl_aob-18028-s01 [file mcy155_suppl_aob-18028-s01.docx]

**Supplementary information**

**Supplementary Data Fig. S1**. Photographs of gravimetric system used for the watering treatments.

**Supplementary Data Fig. S2**. Average number of days for the three miscanthus genotypes to achieve 15% soil water content (SWC) after the start of withholding water.

**Supplementary Data Fig. S3**. Weight measures and derived water content (WC) from tissue harvest.

**Supplementary Data Fig. S4**. Photosynthetic productivity measurements.

**Supplementary data Table S1** – ANOVA for the variation of plant fresh weight measurements.

**Supplementary data Table S2** – ANOVA for the variation of plant dry weight measurements.

**Supplementary data Table S3** – ANOVA for saccharification of leaf and stem biomass.

**Supplementary data Table S4** – Saccharification assays on cell wall material from harvested tissue samples

**Supplementary Data Table S5a** – *P*-values of saccharification differences of leaf biomass, for each combination of genotype, nutrition level and soil water content.

**Supplementary Data Table S5b** – *P*-values of saccharification differences of stem biomass, for each combination of genotype, nutrition level and soil water content.

**Supplementary Data Table S6** – ANOVA matrix monosaccharides from leaf and stem separately.

**Supplementary Data Table S7a** – *P*-values for significant differences in the leaf content of each matrix monosaccharide between genotypes attributed to nutrition level.

**Supplementary Data Table S7b** – *P*-values for significant differences in the stem content of each matrix monosaccharide between genotypes attributed to nutrition level.

**Supplementary data Table S8** – ANOVA cellulose content all samples.

**Supplementary data Table S9** – *P*-values for significant differences in cellulose content between genotypes attributed to nutrition level.

**Supplementary data Table S10** – ANOVA for acetyl bromide lignin determinations.

**Supplementary Data Table S11** – *P*-values for significant differences in lignin content between genotypes attributed to nutrition level.

**Supplementary Data Table S12** – Relative sugar release potential (%) based on saccharification results and dry biomass measures.

**Supplementary Data Table S13**– Pearson’s correlation coefficient (r) between sugar release data and cell wall content.

Supplementary Figure S1


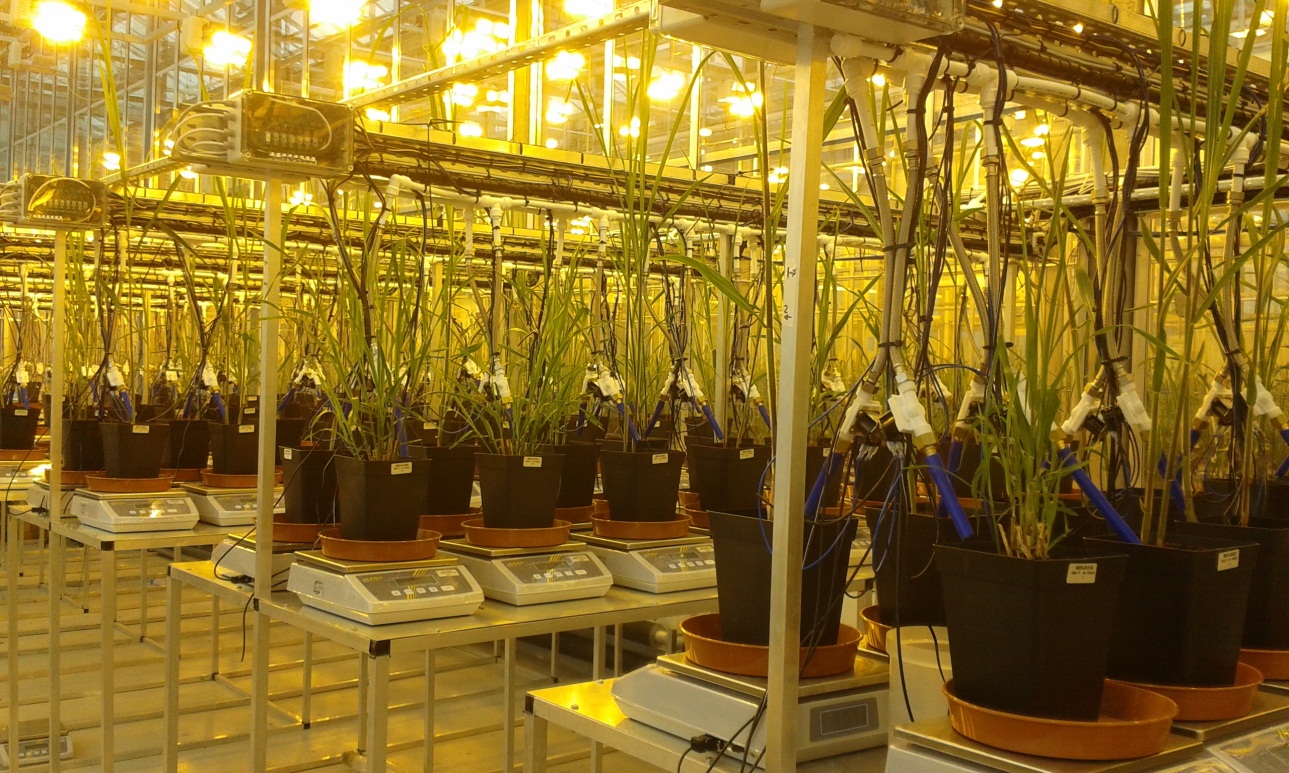

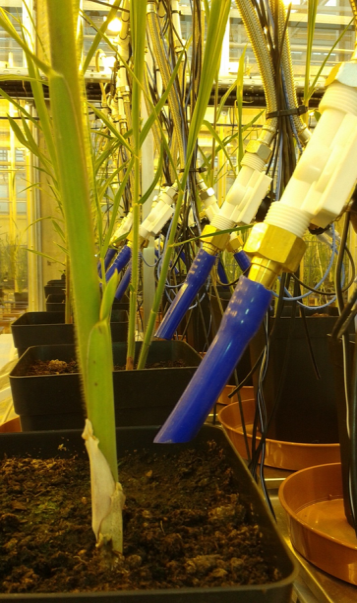

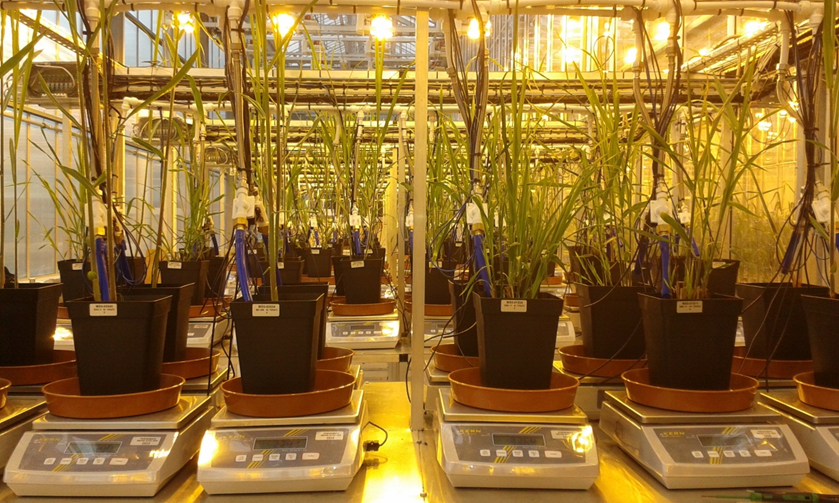


Supplementary Figure S1. Photographs of gravimetric system used for the watering treatments. All plants were potted to defined weights allowing target watering to pre-determined water contents (either 75% or 15% soil water content). Each plant was placed on a watering station which consisted of an electronic balance (Kern FCB12K1), linked via an RS232 serial cable and USB to serial converter to a Raspberry Pi single board computer. Data from 8 balances was collected on each Raspberry Pi. The weight of the plant was monitored at 5 minute intervals and water added twice daily to return the plant to its original target weight. Plants were imaged at weekly intervals using the Lemnatec RGB imaging cabin.

Supplementary Figure S2

**
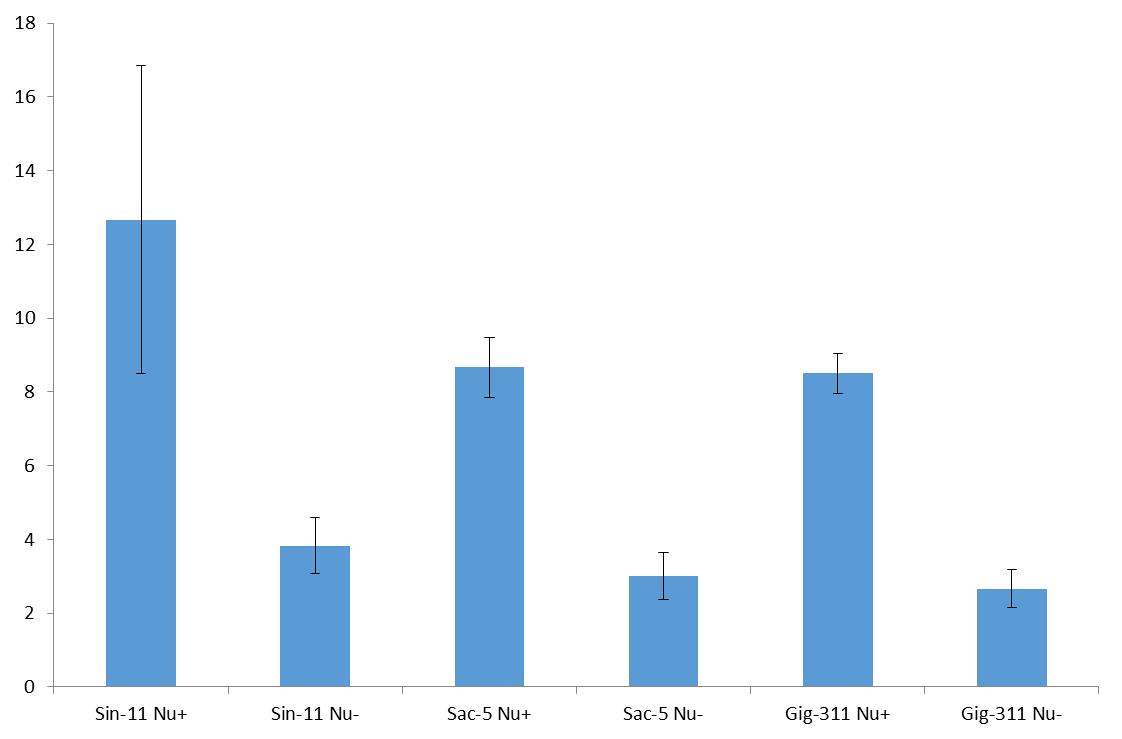
**

Supplementary Figure S2. Average number of days for the three miscanthus genotypes (Sin-11, Sac-5 and Gig-311) to achieve 15% soil water content (SWC) after the start of withholding water (26 days after planting). Nu+, nutrient-rich; Nu-, nutrient-poor. Error bars indicate standard deviation.

Supplementary Figure S3

**
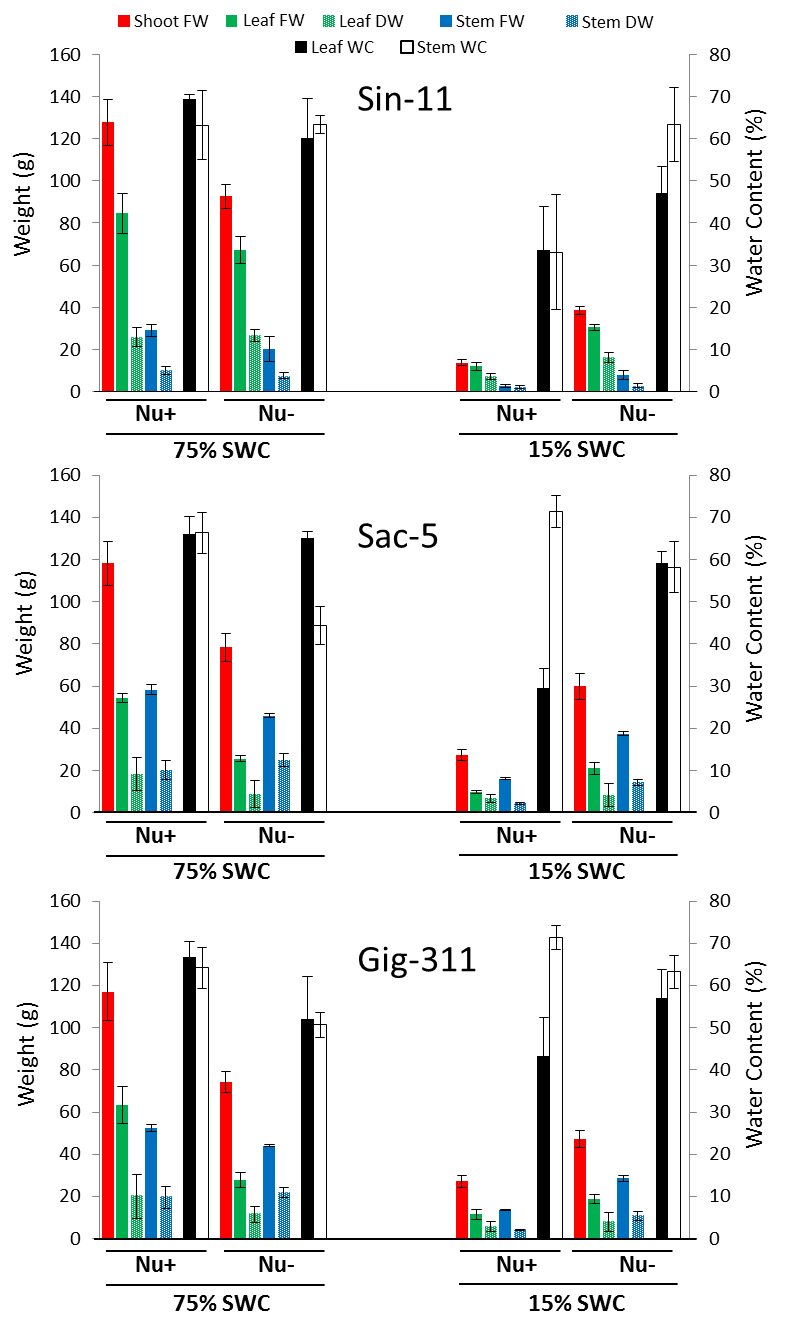
**

Supplementary Figure S3. Weight measures and derived water content (WC) from tissue harvest after the experimental treatments of the three miscanthus genotypes. Weight measures (left y-axis) are in colour coded bars; WC calculations (right y-axis) are in black (leaf) and white (stem) bars. FW is fresh weight, DW is dry weight. Nu+, nutrient-rich; Nu-, nutrient-poor. Weight measures are averages from 5 or 6 biological replicates. Error bars indicate standard error.

Supplementary Figure S4


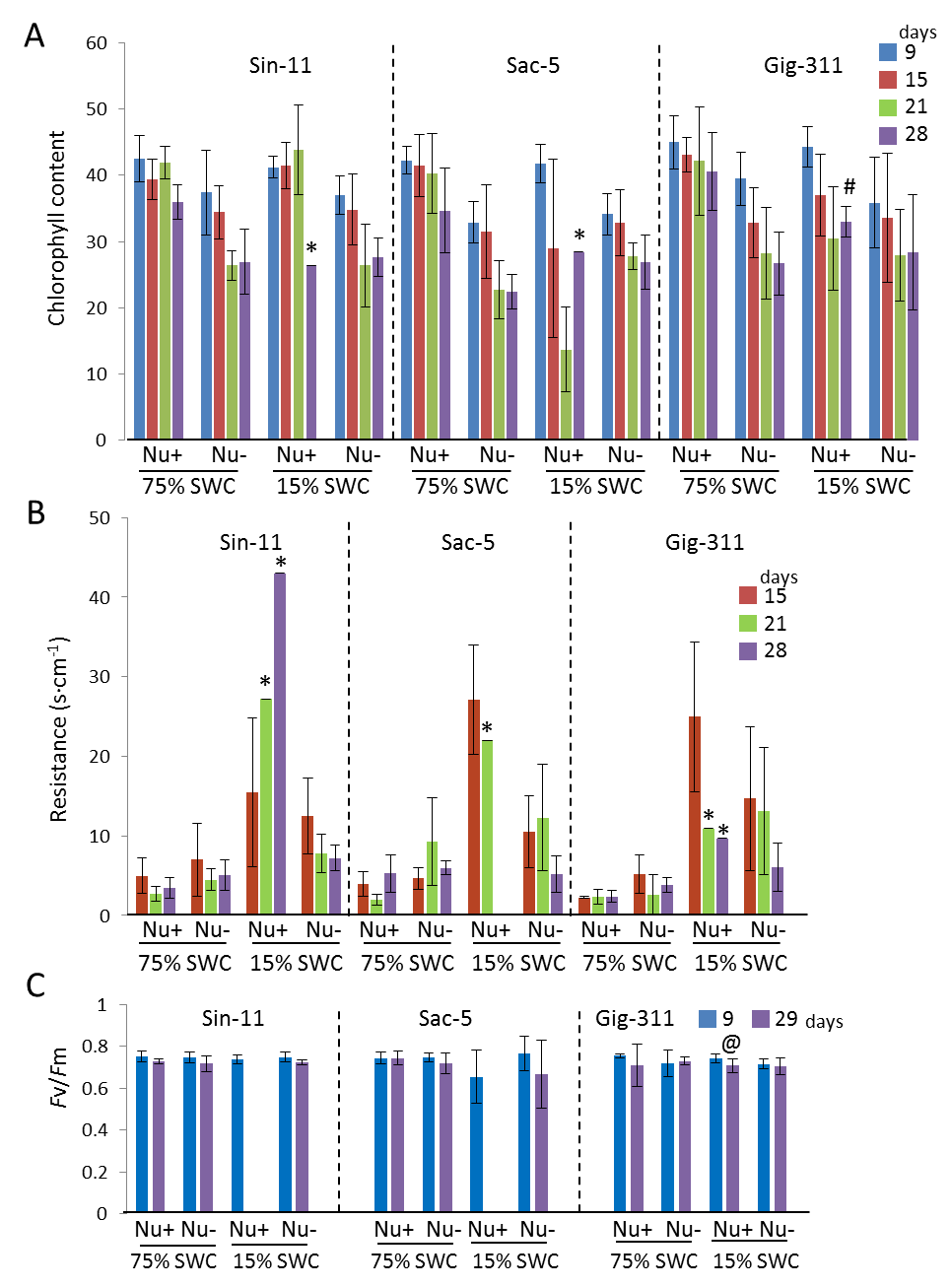
 Supplementary Figure S4. Photosynthetic productivity measurements. A: Average chlorophyll content from 4-6 plants, except * only one plant; # from 3 plants. B: Stomatal conductance from 3-6 plants, except * only one plant. No readings were possible for Nu+ / 15% SWC at 28 days. C: Chlorophyll fluorescence. Maximum quantum yield (Fv/Fm) values from 4-6 plants, except @ readings from 2 plants. No readings could be obtained from Nu+ / 15% SWC for Sin-11 and Sac-5 at day 29. Colour coded bars refer to days after start drought treatments as indicated. Nu+, nutrient-rich; Nu-, nutrient-poor; SWC, soil water content. Error bars indicate standard deviation.

# **Supplementary Table S1**

– ANOVA for the variation of plant fresh weight measurements.

| **Whole plant fresh weight** | | | | | | |
| --- | --- | --- | --- | --- | --- | --- |
| **Effect** | **Sum of squares** | **Degrees of freedom** | **Mean square** | ***F*-ratio** | ***P*-value** | **Effect size (*η^2^*)** |
| Genotype | 0.00 | 2 | 0.00 | 0.41 | 0.669 | 0.002 |
| Nutrition level | 0.00 | 1 | 0.00 | 2.72 | 0.104 | 0.007 |
| Soil Water Content (SWC) | 0.08 | 1 | 0.08 | 260.16 | *0.000* | 0.653 |
| Geno. × Nut. Level. | 0.00 | 2 | 0.00 | 0.34 | 0.712 | 0.002 |
| Geno. × SWC | 0.00 | 2 | 0.00 | 5.06 | *0.009* | 0.025 |
| Nut. level × SWC | 0.02 | 1 | 0.02 | 63.62 | *0.000* | 0.160 |
| Geno × Nutrition × SWC | 0.00 | 2 | 0.00 | 0.21 | 0.808 | 0.001 |
| Error | 0.02 | 60 | 0.00 |  |  |  |
| Total | 0.12 | 71 |  |  |  |  |
| **Leaf fresh weight** | | | | | | |
| **Effect** | **Sum of squares** | **Degrees of freedom** | **Mean square** | ***F*-ratio** | ***P*-value** | **Effect size (*η^2^*)** |
| Genotype | 0.01 | 2 | 0.00 | 23.63 | *0.000* | 0.126 |
| Nutrition level | 0.00 | 1 | 0.00 | 7.53 | *0.008* | 0.020 |
| Soil Water Content (SWC) | 0.02 | 1 | 0.02 | 184.54 | *0.000* | 0.492 |
| Geno. × Nut. Level. | 0.00 | 2 | 0.00 | 2.53 | 0.089 | 0.013 |
| Geno. × SWC | 0.00 | 2 | 0.00 | 11.59 | *0.000* | 0.062 |
| Nut. level × SWC | 0.01 | 1 | 0.01 | 53.98 | *0.000* | 0.144 |
| Geno × Nutrition × SWC | 0.00 | 2 | 0.00 | 0.13 | 0.877 | 0.001 |
| Error | 0.01 | 53 | 0.00 |  |  |  |
| Total | 0.04 | 64 |  |  |  |  |
| **Stem fresh weight** | | | | | | |
| **Effect** | **Sum of squares** | **Degrees of freedom** | **Mean square** | ***F*-ratio** | ***P*-value** | **Effect size (*η^2^*)** |
| Genotype | 0.01 | 2 | 0.00 | 26.53 | *0.000* | 0.270 |
| Nutrition level | 0.00 | 1 | 0.00 | 0.46 | 0.501 | 0.002 |
| Soil Water Content (SWC) | 0.01 | 1 | 0.01 | 68.74 | *0.000* | 0.350 |
| Geno. × Nut. Level. | 0.00 | 2 | 0.00 | 0.44 | 0.647 | 0.004 |
| Geno. × SWC | 0.00 | 2 | 0.00 | 0.67 | 0.515 | 0.007 |
| Nut. level × SWC | 0.00 | 1 | 0.00 | 16.91 | *0.000* | 0.086 |
| Geno × Nutrition × SWC | 0.00 | 2 | 0.00 | 0.94 | 0.397 | 0.010 |
| Error | 0.01 | 53 | 0.00 |  |  |  |
| Total | 0.03 | 64 |  |  |  |  |

**Supplementary Table S2**

– ANOVA for the variation of plant dry weight measurements.

| **Leaf dry weight** | | | | | | |
| --- | --- | --- | --- | --- | --- | --- |
| **Effect** | **Sum of squares** | **Degrees of freedom** | **Mean square** | ***F*-ratio** | ***P*-value** | **Effect size (*η^2^*)** |
| Genotype | 0.00 | 2 | 0.00 | 18.28 | *0.000* | 0.195 |
| Nutrition level | 0.00 | 1 | 0.00 | 0.33 | 0.566 | 0.002 |
| Soil Water Content (SWC) | 0.00 | 1 | 0.00 | 63.09 | *0.000* | 0.337 |
| Geno. × Nut. Level. | 0.00 | 2 | 0.00 | 5.06 | *0.010* | 0.054 |
| Geno. × SWC | 0.00 | 2 | 0.00 | 3.92 | *0.026* | 0.042 |
| Nut. level × SWC | 0.00 | 1 | 0.00 | 16.22 | *0.000* | 0.087 |
| Geno × Nutrition × SWC | 0.00 | 2 | 0.00 | 0.12 | 0.884 | 0.001 |
| Error | 0.00 | 53 | 0.00 |  |  |  |
| Total | 0.01 | 64 |  |  |  |  |
| **Stem dry weight** | | | | | | |
| **Effect** | **Sum of squares** | **Degrees of freedom** | **Mean square** | ***F*-ratio** | ***P*-value** | **Effect size (*η^2^*)** |
| Genotype | 0.00 | 2 | 0.00 | 20.60 | *0.000* | 0.234 |
| Nutrition level | 0.00 | 1 | 0.00 | 6.98 | *0.011* | 0.040 |
| Soil Water Content (SWC) | 0.00 | 1 | 0.00 | 62.14 | *0.000* | 0.352 |
| Geno. × Nut. Level. | 0.00 | 2 | 0.00 | 2.84 | 0.068 | 0.032 |
| Geno. × SWC | 0.00 | 2 | 0.00 | 2.43 | 0.098 | 0.028 |
| Nut. level × SWC | 0.00 | 1 | 0.00 | 2.50 | 0.120 | 0.014 |
| Geno × Nutrition × SWC | 0.00 | 2 | 0.00 | 0.02 | 0.976 | 0.000 |
| Error | 0.00 | 53 | 0.00 |  |  |  |
| Total | 0.01 | 64 |  |  |  |  |

**Supplementary Table S3**

– ANOVA for saccharification of leaf and stem biomass.

| **Leaf** | | | | | | |
| --- | --- | --- | --- | --- | --- | --- |
| **Effect** | **Sum of squares** | **Degrees of freedom** | **Mean square** | ***F*-ratio** | ***P*-value** | **Effect size (*η^2^*)** |
| **Genotype** | 413.90 | 2 | 207.00 | 4.02 | ***0.020*** | 0.037 |
| **Nutrition level** | 428.30 | 1 | 428.30 | 8.32 | ***0.005*** | 0.038 |
| **SWC** | 813.20 | 1 | 813.20 | 15.80 | ***≤0.001*** | 0.072 |
| **Geno. × Nut. Level.** | 187.20 | 2 | 93.60 | 1.82 | 0.166 | 0.017 |
| **Geno. × SWC** | 367.20 | 2 | 183.60 | 3.57 | ***0.031*** | 0.032 |
| **Nutrition × SWC** | 1363.90 | 1 | 1363.90 | 26.50 | ***≤0.001*** | 0.120 |
| **Geno. × Nut. × SWC** | 286.00 | 2 | 143.00 | 2.78 | 0.065 | 0.025 |
| **Error** | 7463.70 | 145 | 51.50 |  |  |  |
| **Total** | 11323.40 | 156 |  |  |  |  |
|  |  |  |  |  |  |  |
| **Stem** | | | | | | |
| **Effect** | **Sum of squares** | **Degrees of freedom** | **Mean square** | ***F*-ratio** | ***P*-value** | **Effect size (*η^2^*)** |
| **Genotype** | 1542.00 | 2 | 771.00 | 22.72 | ***≤0.001*** | 0.157 |
| **Nutrition level** | 266.20 | 1 | 266.20 | 7.84 | ***0.006*** | 0.027 |
| **SWC** | 1631.10 | 1 | 1631.10 | 48.06 | ***≤0.001*** | 0.166 |
| **Geno. × Nut. Level.** | 458.30 | 2 | 229.10 | 6.75 | ***0.002*** | 0.047 |
| **Geno. × SWC** | 516.40 | 2 | 258.20 | 7.61 | ***0.001*** | 0.053 |
| **Nutrition × SWC** | 938.50 | 1 | 938.50 | 27.65 | ***≤0.001*** | 0.096 |
| **Geno. × Nut. × SWC** | 628.40 | 2 | 314.20 | 9.26 | ***≤0.001*** | 0.064 |
| **Error** | 3835.40 | 113 | 33.90 |  |  |  |
| **Total** | 9816.30 | 124 |  |  |  |  |

# **Supplementary Table S4**

– Saccharification assays on cell wall material from harvested leaf and stem samples

| **Reducing Sugar Equivalent** | | | | | | | | | | |
| --- | --- | --- | --- | --- | --- | --- | --- | --- | --- | --- |
|  |  |  |  | **Leaf** | | |  | **Stem** | | |
|  |  | **SWC** |  | **Nutrient-poor** |  | **Nutrient-rich** |  | **Nutrient-poor** |  | **Nutrient-rich** |
| **Sin-11** |  | **15%** |  | 44.08 ± 4.04 |  | 44.91 ± 5.77 |  | 45.24 ± 6.29 |  | 53.88 ± 5.86 |
|  |  | **75%** |  | 48.78 ± 6.04 |  | 45.39 ± 5.84 |  | 48.59 ± 11.09 |  | 35.63 ± 3.84 |
|  |  |  |  |  |  |  |  |  |  |  |
| **Sac-5** |  | **15%** |  | 42.04 ± 5.07 |  | 47.26 ± 4.87 |  | 38.81 ± 4.24 |  | 45.44 ± 2.80 |
|  |  | **75%** |  | 58.49 ± 11.23 |  | 48.65 ± 8.81 |  | 36.99 ± 4.36 |  | 43.08 ± 2.91 |
|  |  |  |  |  |  |  |  |  |  |  |
| **Gig-311** |  | **15%** |  | 43.53 ± 11.22 |  | 45.30 ± 4.53 |  | 38.07 ± 3.34 |  | 48.47 ± 10.74 |
|  |  | **75%** |  | 53.93 ± 8.81 |  | 39.37 ± 3.53 |  | 31.21 ± 2.70 |  | 30.24 ± 2.29 |

The cell wall biomass was subjected to a mild pretreatment (water at 94°C for 30 min) before enzymatic saccharification. Sugar release was measured by colorimetric detection of reducing sugar equivalents. Values are mean ± standard deviation of the biological and technical replicates (minimum for each value is n=7, range 7 – 15; total samples is n=350). Outliers excluded via the z-score calculation method (excluded z-score>4). The bar graph of the saccharification data is shown in Figure 4.

# **Supplementary Table S5a**

– *P*-values of saccharification differences of leaf biomass, for each combination of genotype, nutrition level and soil water content (Tukey test of the interaction Geno. × Nut. Level). w, water content.

| **Leaf** | | | | | | | | | | | | |
| --- | --- | --- | --- | --- | --- | --- | --- | --- | --- | --- | --- | --- |
|  | **Sin-11 Nu- w15** | **Sin-11 Nu- w75** | **Sin-11 Nu+ w15** | **Sin-11 Nu+ w75** | **Gig-311 Nu- w15** | **Gig-311 Nu- w75** | **Gig-311 Nu+ w15** | **Gig-311 Nu+ w75** | **Sac-5 Nu- w15** | **Sac-5 Nu- w75** | **Sac-5 Nu+ w15** | **Sac-5 Nu+ w75** |
| **Sin-11 Nu- w15** |  | 0.8821 | 1.0000 | 1.0000 | 1.0000 | ***0.0155*** | 1.0000 | 0.8807 | 0.9999 | ***0.0001*** | 0.9922 | 0.8773 |
| **Sin-11 Nu- w75** | 0.8821 |  | 0.9731 | 0.9871 | 0.7588 | 0.7641 | 0.9921 | ***0.0396*** | 0.4095 | ***0.0446*** | 1.0000 | 1.0000 |
| **Sin-11 Nu+ w15** | 1.0000 | 0.9731 |  | 1.0000 | 1.0000 | 0.0540 | 1.0000 | 0.7408 | 0.9977 | ***0.0004*** | 0.9996 | 0.9732 |
| **Sin-11 Nu+ w75** | 1.0000 | 0.9871 | 1.0000 |  | 0.9999 | 0.0615 | 1.0000 | 0.5650 | 0.9880 | ***0.0004*** | 0.9999 | 0.9874 |
| **Gig-311 Nu- w15** | 1.0000 | 0.7588 | 1.0000 | 0.9999 |  | ***0.0054*** | 1.0000 | 0.9403 | 1.0000 | ***≤0.0001*** | 0.9678 | 0.7455 |
| **Gig-311 Nu- w75** | ***0.0155*** | 0.7641 | 0.0540 | 0.0615 | ***0.0054*** |  | 0.1250 | ***≤0.0001*** | ***0.0008*** | 0.9082 | 0.3405 | 0.6844 |
| **Gig-311 Nu+ w15** | 1.0000 | 0.9921 | 1.0000 | 1.0000 | 1.0000 | 0.1250 |  | 0.7188 | 0.9956 | ***0.0016*** | 1.0000 | 0.9926 |
| **Gig-311 Nu+ w75** | 0.8807 | ***0.0396*** | 0.7408 | 0.5650 | 0.9403 | ***≤0.0001*** | 0.7188 |  | 0.9986 | ***≤0.0001*** | 0.1573 | ***0.0316*** |
| **Sac-5 Nu- w15** | 0.9999 | 0.4095 | 0.9977 | 0.9880 | 1.0000 | ***0.0008*** | 0.9956 | 0.9986 |  | ***≤0.0001*** | 0.7659 | 0.3847 |
| **Sac-5 Nu- w75** | ***0.0001*** | ***0.0446*** | ***0.0004*** | ***0.0004*** | ***≤0.0001*** | 0.9082 | ***0.0016*** | ***≤0.0001*** | ***≤0.0001*** |  | ***0.0058*** | ***0.0274*** |
| **Sac-5 Nu+ w15** | 0.9922 | 1.0000 | 0.9996 | 0.9999 | 0.9678 | 0.3405 | 1.0000 | 0.1573 | 0.7659 | ***0.0058*** |  | 1.0000 |
| **Sac-5 Nu+ w75** | 0.8773 | 1.0000 | 0.9732 | 0.9874 | 0.7455 | 0.6844 | 0.9926 | ***0.0316*** | 0.3847 | ***0.0274*** | 1.0000 |  |

# Supplementary Table S5b

– *P*-values of saccharification differences of stem biomass, for each combination of genotype, nutrition level and soil water content (Tukey test of the interaction Geno. × Nut. Level). w, water content.

| **Stem** | | | | | | | | | | | | |
| --- | --- | --- | --- | --- | --- | --- | --- | --- | --- | --- | --- | --- |
|  | **Sin-11 Nu- w15** | **Sin-11 Nu- w75** | **Sin-11 Nu+ w15** | **Sin-11 Nu+ w75** | **Gig-311 Nu- w15** | **Gig-311 Nu- w75** | **Gig-311 Nu+ w15** | **Gig-311 Nu+ w75** | **Sac-5 Nu- w15** | **Sac-5 Nu- w75** | **Sac-5 Nu+ w15** | **Sac-5 Nu+ w75** |
| **Sin-11 Nu- w15** |  | 0.9395 | ***0.0243*** | ***0.0045*** | 0.1633 | ***0.0001*** | 0.9881 | ***0.0001*** | 0.2574 | ***0.0396*** | 1.0000 | 0.9986 |
| **Sin-11 Nu- w75** | 0.9395 |  | 0.5829 | ***0.0001*** | ***0.0036*** | ***0.0001*** | 1.0000 | ***0.0001*** | ***0.0064*** | ***0.0005*** | 0.9705 | 0.4416 |
| **Sin-11 Nu+ w15** | ***0.0243*** | 0.5829 |  | ***0.0001*** | ***0.0001*** | ***0.0001*** | 0.7669 | ***0.0001*** | ***0.0001*** | ***0.0001*** | ***0.0442*** | ***0.0020*** |
| **Sin-11 Nu+ w75** | ***0.0045*** | ***0.0001*** | ***0.0001*** |  | 0.9987 | 0.8700 | ***0.0009*** | 0.7003 | 0.9835 | 1.0000 | ***0.0055*** | 0.1037 |
| **Gig-311 Nu- w15** | 0.1633 | ***0.0036*** | ***0.0001*** | 0.9987 |  | 0.3507 | ***0.0275*** | 0.2094 | 1.0000 | 1.0000 | 0.1669 | 0.7250 |
| **Gig-311 Nu- w75** | ***0.0001*** | ***0.0001*** | ***0.0001*** | 0.8700 | 0.3507 |  | ***0.0001*** | 1.0000 | 0.1782 | 0.5812 | ***0.0001*** | ***0.0007*** |
| **Gig-311 Nu+ w15** | 0.9881 | 1.0000 | 0.7669 | ***0.0009*** | ***0.0275*** | ***0.0001*** |  | ***0.0001*** | ***0.0462*** | ***0.0062*** | 0.9945 | 0.7292 |
| **Gig-311 Nu+ w75** | ***0.0001*** | ***0.0001*** | ***0.0001*** | 0.7003 | 0.2094 | 1.0000 | ***0.0001*** |  | 0.0956 | 0.3881 | ***0.0001*** | ***0.0004*** |
| **Sac-5 Nu- w15** | 0.2574 | ***0.0064*** | ***0.0001*** | 0.9835 | 1.0000 | 0.1782 | ***0.0462*** | 0.0956 |  | 0.9999 | 0.2603 | 0.8579 |
| **Sac-5 Nu- w75** | ***0.0396*** | ***0.0005*** | ***0.0001*** | 1.0000 | 1.0000 | 0.5812 | ***0.0062*** | 0.3881 | 0.9999 |  | ***0.0433*** | 0.3868 |
| **Sac-5 Nu+ w15** | 1.0000 | 0.9705 | ***0.0442*** | ***0.0055*** | 0.1669 | ***0.0001*** | 0.9945 | ***0.0001*** | 0.2603 | ***0.0433*** |  | 0.9977 |
| **Sac-5 Nu+ w75** | 0.9986 | 0.4416 | ***0.0020*** | 0.1037 | 0.7250 | ***0.0007*** | 0.7292 | ***0.0004*** | 0.8579 | 0.3868 | 0.9977 |  |

# Supplementary Table S6

– ANOVA matrix monosaccharides from leaf and stem separately.

|  | **Effect** |  | **Sum of squares** | **Degrees of freedom** | **Mean square** | ***F*-ratio** | ***P*-value** | **Effect size (*η^2^*)** | | **Sum of squares** | **Degrees of freedom** | **Mean square** | ***F*-ratio** | ***P*-value** | **Effect size (*η^2^*)** |
| --- | --- | --- | --- | --- | --- | --- | --- | --- | --- | --- | --- | --- | --- | --- | --- |
|  |  |  |  |  |  | **Leaf** |  |  |  |  |  |  | **Stem** |  |  |
|  |  |  |  |  |  |  |  |  |  |  |  |  |  |  |  |
|  | Geno |  | 0.000 | 2 | 0.000 | 2.05 | 0.158 | 0.143 |  | 0.000 | 2 | 0.000 | 2.83 | 0.085 | 0.220 |
|  | Nutrition |  | 0.000 | 1 | 0.000 | 4.96 | ***0.039*** | 0.172 |  | 0.000 | 1 | 0.000 | 0.32 | 0.579 | 0.012 |
| **Fuc** | Geno×Nutrition |  | 0.000 | 2 | 0.000 | 0.81 | 0.460 | 0.057 |  | 0.000 | 2 | 0.000 | 0.92 | 0.417 | 0.071 |
|  | Error |  | 0.000 | 18 | 0.000 |  |  |  |  | 0.001 | 18 | 0.000 |  |  |  |
|  | Total |  | 0.001 | 23 |  |  |  |  |  | 0.001 | 23 |  |  |  |  |
|  |  |  |  |  |  |  |  |  |  |  |  |  |  |  |  |
|  | Geno |  | 1.437 | 2 | 0.718 | 10.96 | ***0.001*** | 0.505 |  | 7.641 | 2 | 3.820 | 17.82 | ***<0.001*** | 0.629 |
|  | Nutrition |  | 0.044 | 1 | 0.044 | 0.68 | 0.422 | 0.016 |  | 0.134 | 1 | 0.134 | 0.62 | 0.440 | 0.011 |
| **Ara** | Geno×Nutrition |  | 0.182 | 2 | 0.091 | 1.39 | 0.274 | 0.064 |  | 0.509 | 2 | 0.254 | 1.19 | 0.328 | 0.042 |
|  | Error |  | 1.180 | 18 | 0.066 |  |  |  |  | 3.859 | 18 | 0.214 |  |  |  |
|  | Total |  | 2.844 | 23 |  |  |  |  |  | 12.141 | 23 |  |  |  |  |
|  |  |  |  |  |  |  |  |  |  |  |  |  |  |  |  |
|  | Geno |  | 0.286 | 2 | 0.143 | 29.83 | ***<0.001*** | 0.731 |  | 0.362 | 2 | 0.181 | 11.27 | ***0.001*** | 0.490 |
|  | Nutrition |  | 0.004 | 1 | 0.004 | 0.79 | 0.386 | 0.010 |  | 0.033 | 1 | 0.033 | 2.03 | 0.171 | 0.044 |
| **Gal** | Geno×Nutrition |  | 0.015 | 2 | 0.007 | 1.56 | 0.238 | 0.038 |  | 0.054 | 2 | 0.027 | 1.69 | 0.212 | 0.074 |
|  | Error |  | 0.086 | 18 | 0.005 |  |  |  |  | 0.290 | 18 | 0.016 |  |  |  |
|  | Total |  | 0.391 | 23 |  |  |  |  |  | 0.739 | 23 |  |  |  |  |
|  |  |  |  |  |  |  |  |  |  |  |  |  |  |  |  |
|  | Geno |  | 0.000 | 2 | 0.000 | 0.71 | 0.505 | 0.062 |  | 0.001 | 2 | 0.001 | 6.45 | ***0.008*** | 0.320 |
|  | Nutrition |  | 0.000 | 1 | 0.000 | 0.20 | 0.661 | 0.009 |  | 0.000 | 1 | 0.000 | 2.27 | 0.149 | 0.056 |
| **Rha** | Geno×Nutrition |  | 0.000 | 2 | 0.000 | 1.56 | 0.237 | 0.137 |  | 0.001 | 2 | 0.000 | 3.57 | ***0.050*** | 0.177 |
|  | Error |  | 0.002 | 18 | 0.000 |  |  |  |  | 0.002 | 18 | 0.000 |  |  |  |
|  | Total |  | 0.003 | 23 |  |  |  |  |  | 0.004 | 23 |  |  |  |  |
|  |  |  |  |  |  |  |  |  |  |  |  |  |  |  |  |
|  | Geno |  | 58.403 | 2 | 29.201 | 4.42 | ***0.027*** | 0.269 |  | 10.289 | 2 | 5.144 | 0.34 | 0.716 | 0.017 |
|  | Nutrition |  | 24.833 | 1 | 24.833 | 3.76 | 0.068 | 0.114 |  | 222.292 | 1 | 222.292 | 14.70 | ***0.001*** | 0.373 |
| **Glc** | Geno×Nutrition |  | 14.660 | 2 | 7.330 | 1.11 | 0.352 | 0.068 |  | 90.874 | 2 | 45.437 | 3.00 | 0.075 | 0.153 |
|  | Error |  | 119.001 | 18 | 6.611 |  |  |  |  | 272.276 | 18 | 15.126 |  |  |  |
|  | Total |  | 216.896 | 23 |  |  |  |  |  | 595.731 | 23 |  |  |  |  |
|  |  |  |  |  |  |  |  |  |  |  |  |  |  |  |  |
|  | Geno |  | 0.324 | 2 | 0.162 | 0.12 | 0.891 | 0.012 |  | 93.957 | 2 | 46.978 | 34.26 | ***<0.001*** | 0.733 |
|  | Nutrition |  | 0.008 | 1 | 0.008 | 0.01 | 0.939 | 0.000 |  | 4.098 | 1 | 4.098 | 2.99 | 0.101 | 0.032 |
| **Xyl** | Geno×Nutrition |  | 1.217 | 2 | 0.609 | 0.44 | 0.652 | 0.046 |  | 5.384 | 2 | 2.692 | 1.96 | 0.169 | 0.042 |
|  | Error |  | 25.036 | 18 | 1.391 |  |  |  |  | 24.682 | 18 | 1.371 |  |  |  |
|  | Total |  | 26.585 | 23 |  |  |  |  |  | 128.121 | 23 |  |  |  |  |
|  |  |  |  |  |  |  |  |  |  |  |  |  |  |  |  |
|  | Geno |  | 0.000 | 2 | 0.000 | 0.03 | 0.966 | 0.003 |  | 0.004 | 2 | 0.002 | 8.96 | ***0.002*** | 0.445 |
|  | Nutrition |  | 0.001 | 1 | 0.001 | 2.03 | 0.171 | 0.084 |  | 0.000 | 1 | 0.000 | 1.01 | 0.329 | 0.025 |
| **Man** | Geno×Nutrition |  | 0.002 | 2 | 0.001 | 1.97 | 0.169 | 0.164 |  | 0.001 | 2 | 0.000 | 1.68 | 0.215 | 0.083 |
|  | Error |  | 0.008 | 18 | 0.000 |  |  |  |  | 0.004 | 18 | 0.000 |  |  |  |
|  | Total |  | 0.011 | 23 |  |  |  |  |  | 0.008 | 23 |  |  |  |  |
|  |  |  |  |  |  |  |  |  |  |  |  |  |  |  |  |

#

# Supplementary Table S7a

– *P*-values for significant differences in the leaf content of each matrix monosaccharide between genotypes attributed to nutrition level (Tukey test of the interaction Geno. × Nut. Level).

|  |  |  |  |  | **Leaf** |  |  |  |
| --- | --- | --- | --- | --- | --- | --- | --- | --- |
|  |  |  | **Sin-11 Nu-** | **Sin-11 Nu+** | **Gig-311 Nu-** | **Gig-311 Nu+** | **Sac-5 Nu-** | **Sac-5 Nu+** |
|  |  |  |  |  |  |  |  |  |
|  | **Sin-11 Nu-** |  |  | 0.8847 | 0.6480 | 0.3585 | 0.9874 | 0.0856 |
|  | **Sin-11 Nu+** |  | 0.8847 |  | 0.9971 | 0.9228 | 0.9974 | 0.4758 |
| **Fuc** | **Gig-311 Nu-** |  | 0.6480 | 0.9971 |  | 0.9953 | 0.9398 | 0.7458 |
|  | **Gig-311 Nu+** |  | 0.3585 | 0.9228 | 0.9953 |  | 0.7186 | 0.9517 |
|  | **Sac-5 Nu-** |  | 0.9874 | 0.9974 | 0.9398 | 0.7186 |  | 0.2550 |
|  | **Sac-5 Nu+** |  | 0.0856 | 0.4758 | 0.7458 | 0.9517 | 0.2550 |  |
|  |  |  |  |  |  |  |  |  |
|  | **Sin-11 Nu-** |  |  | 0.9546 | 0.1054 | 0.4100 | 0.0704 | ***0.0038*** |
|  | **Sin-11 Nu+** |  | 0.9546 |  | 0.4098 | 0.8762 | 0.3037 | ***0.0222*** |
| **Ara** | **Gig-311 Nu-** |  | 0.1054 | 0.4098 |  | 0.9545 | 0.9999 | 0.5860 |
|  | **Gig-311 Nu+** |  | 0.4100 | 0.8762 | 0.9545 |  | 0.8906 | 0.1808 |
|  | **Sac-5 Nu-** |  | 0.0704 | 0.3037 | 0.9999 | 0.8906 |  | 0.7132 |
|  | **Sac-5 Nu+** |  | ***0.0038*** | ***0.0222*** | 0.5860 | 0.1808 | 0.7132 |  |
|  |  |  |  |  |  |  |  |  |
|  | **Sin-11 Nu-** |  |  | 0.6584 | ***0.0020*** | ***0.0122*** | ***0.0004*** | ***0.0002*** |
|  | **Sin-11 Nu+** |  | 0.6584 |  | ***0.0444*** | 0.2241 | ***0.0067*** | ***0.0011*** |
| **Gal** | **Gig-311 Nu-** |  | ***0.0020*** | ***0.0444*** |  | 0.9454 | 0.9368 | 0.4762 |
|  | **Gig-311 Nu+** |  | ***0.0122*** | 0.2241 | 0.9454 |  | 0.4884 | 0.1220 |
|  | **Sac-5 Nu-** |  | ***0.0004*** | ***0.0067*** | 0.9368 | 0.4884 |  | 0.9400 |
|  | **Sac-5 Nu+** |  | ***0.0002*** | ***0.0011*** | 0.4762 | 0.1220 | 0.9400 |  |
|  |  |  |  |  |  |  |  |  |
|  | **Sin-11 Nu-** |  |  | 0.8730 | 0.3469 | 0.9774 | 0.7770 | 0.9676 |
|  | **Sin-11 Nu+** |  | 0.8730 |  | 0.9246 | 0.9987 | 0.9999 | 0.9995 |
| **Rha** | **Gig-311 Nu-** |  | 0.3469 | 0.9246 |  | 0.7549 | 0.9713 | 0.7887 |
|  | **Gig-311 Nu+** |  | 0.9774 | 0.9987 | 0.7549 |  | 0.9903 | 1.0000 |
|  | **Sac-5 Nu-** |  | 0.7770 | 0.9999 | 0.9713 | 0.9903 |  | 0.9943 |
|  | **Sac-5 Nu+** |  | 0.9676 | 0.9995 | 0.7887 | 1.0000 | 0.9943 |  |
|  |  |  |  |  |  |  |  |  |
|  | **Sin-11 Nu-** |  |  | 0.8038 | 0.1488 | 0.1477 | 0.8325 | ***0.0398*** |
|  | **Sin-11 Nu+** |  | 0.8038 |  | 0.7596 | 0.7574 | 1.0000 | 0.3609 |
| **Glc** | **Gig-311 Nu-** |  | 0.1488 | 0.7596 |  | 1.0000 | 0.7268 | 0.9800 |
|  | **Gig-311 Nu+** |  | 0.1477 | 0.7574 | 1.0000 |  | 0.7246 | 0.9805 |
|  | **Sac-5 Nu-** |  | 0.8325 | 1.0000 | 0.7268 | 0.7246 |  | 0.3320 |
|  | **Sac-5 Nu+** |  | ***0.0398*** | 0.3609 | 0.9800 | 0.9805 | 0.3320 |  |
|  |  |  |  |  |  |  |  |  |
|  | **Sin-11 Nu-** |  |  | 0.9997 | 0.9981 | 0.9987 | 0.9987 | 1.0000 |
|  | **Sin-11 Nu+** |  | 0.9997 |  | 1.0000 | 0.9825 | 0.9827 | 0.9997 |
| **Xyl** | **Gig-311 Nu-** |  | 0.9981 | 1.0000 |  | 0.9626 | 0.9630 | 0.9981 |
|  | **Gig-311 Nu+** |  | 0.9987 | 0.9825 | 0.9626 |  | 1.0000 | 0.9987 |
|  | **Sac-5 Nu-** |  | 0.9987 | 0.9827 | 0.9630 | 1.0000 |  | 0.9988 |
|  | **Sac-5 Nu+** |  | 1.0000 | 0.9997 | 0.9981 | 0.9987 | 0.9988 |  |
|  |  |  |  |  |  |  |  |  |
|  | **Sin-11 Nu-** |  |  | 0.9555 | 0.9901 | 1.0000 | 0.9876 | 0.6147 |
|  | **Sin-11 Nu+** |  | 0.9555 |  | 0.9999 | 0.9568 | 0.6883 | 0.9727 |
| **Man** | **Gig-311 Nu-** |  | 0.9901 | 0.9999 |  | 0.9905 | 0.8213 | 0.9138 |
|  | **Gig-311 Nu+** |  | 1.0000 | 0.9568 | 0.9905 |  | 0.9871 | 0.6182 |
|  | **Sac-5 Nu-** |  | 0.9876 | 0.6883 | 0.8213 | 0.9871 |  | 0.2784 |
|  | **Sac-5 Nu+** |  | 0.6147 | 0.9727 | 0.9138 | 0.6182 | 0.2784 |  |
|  |  |  |  |  |  |  |  |  |

# Supplementary Table S7b

– *P*-values for significant differences in the stem content of each matrix monosaccharide between genotypes attributed to nutrition level (Tukey test of the interaction Geno. × Nut. Level).

|  |  |  |  |  | **Stem** |  |  |  |
| --- | --- | --- | --- | --- | --- | --- | --- | --- |
|  |  |  | **Sin-11 Nu-** | **Sin-11 Nu+** | **Gig-311 Nu-** | **Gig-311 Nu+** | **Sac-5 Nu-** | **Sac-5 Nu+** |
|  |  |  |  |  |  |  |  |  |
|  | **Sin-11 Nu-** |  |  | 0.9991 | 0.9903 | 0.4548 | 0.2991 | 0.6571 |
|  | **Sin-11 Nu+** |  | 0.9991 |  | 0.9999 | 0.6666 | 0.4843 | 0.8490 |
| **Fuc** | **Gig-311 Nu-** |  | 0.9903 | 0.9999 |  | 0.7976 | 0.6239 | 0.9344 |
|  | **Gig-311 Nu+** |  | 0.4548 | 0.6666 | 0.7976 |  | 0.9996 | 0.9993 |
|  | **Sac-5 Nu-** |  | 0.2991 | 0.4843 | 0.6239 | 0.9996 |  | 0.9854 |
|  | **Sac-5 Nu+** |  | 0.6571 | 0.8490 | 0.9344 | 0.9993 | 0.9854 |  |
|  |  |  |  |  |  |  |  |  |
|  | **Sin-11 Nu-** |  |  | 0.9860 | 0.0849 | ***0.0045*** | ***0.0027*** | ***0.0117*** |
|  | **Sin-11 Nu+** |  | 0.9860 |  | 0.2593 | ***0.0173*** | ***0.0100*** | ***0.0435*** |
| **Ara** | **Gig-311 Nu-** |  | 0.0849 | 0.2593 |  | 0.7041 | 0.5476 | 0.9162 |
|  | **Gig-311 Nu+** |  | ***0.0045*** | ***0.0173*** | 0.7041 |  | 0.9998 | 0.9973 |
|  | **Sac-5 Nu-** |  | ***0.0027*** | ***0.0100*** | 0.5476 | 0.9998 |  | 0.9776 |
|  | **Sac-5 Nu+** |  | ***0.0117*** | ***0.0435*** | 0.9162 | 0.9973 | 0.9776 |  |
|  |  |  |  |  |  |  |  |  |
|  | **Sin-11 Nu-** |  |  | 0.4778 | 0.3261 | ***0.0319*** | ***0.0028*** | ***0.0106*** |
|  | **Sin-11 Nu+** |  | 0.4778 |  | 0.9997 | 0.6194 | 0.1102 | 0.3229 |
| **Gal** | **Gig-311 Nu-** |  | 0.3261 | 0.9997 |  | 0.7842 | 0.1830 | 0.4738 |
|  | **Gig-311 Nu+** |  | ***0.0319*** | 0.6194 | 0.7842 |  | 0.8397 | 0.9940 |
|  | **Sac-5 Nu-** |  | ***0.0028*** | 0.1102 | 0.1830 | 0.8397 |  | 0.9854 |
|  | **Sac-5 Nu+** |  | ***0.0106*** | 0.3229 | 0.4738 | 0.9940 | 0.9854 |  |
|  |  |  |  |  |  |  |  |  |
|  | **Sin-11 Nu-** |  |  | 0.7822 | 0.9481 | 0.3450 | 0.0897 | 0.9990 |
|  | **Sin-11 Nu+** |  | 0.7822 |  | 0.2971 | ***0.0343*** | ***0.0065*** | 0.5694 |
| **Rha** | **Gig-311 Nu-** |  | 0.9481 | 0.2971 |  | 0.8333 | 0.3790 | 0.9953 |
|  | **Gig-311 Nu+** |  | 0.3450 | ***0.0343*** | 0.8333 |  | 0.9627 | 0.5474 |
|  | **Sac-5 Nu-** |  | 0.0897 | ***0.0065*** | 0.3790 | 0.9627 |  | 0.1734 |
|  | **Sac-5 Nu+** |  | 0.9990 | 0.5694 | 0.9953 | 0.5474 | 0.1734 |  |
|  |  |  |  |  |  |  |  |  |
|  | **Sin-11 Nu-** |  |  | 0.0633 | 0.4503 | 0.3354 | 0.9986 | ***0.0155*** |
|  | **Sin-11 Nu+** |  | 0.0633 |  | 0.8366 | 0.9220 | 0.1322 | 0.9799 |
| **Glc** | **Gig-311 Nu-** |  | 0.4503 | 0.8366 |  | 0.9999 | 0.6831 | 0.4409 |
|  | **Gig-311 Nu+** |  | 0.3354 | 0.9220 | 0.9999 |  | 0.5517 | 0.5687 |
|  | **Sac-5 Nu-** |  | 0.9986 | 0.1322 | 0.6831 | 0.5517 |  | ***0.0347*** |
|  | **Sac-5 Nu+** |  | ***0.0155*** | 0.9799 | 0.4409 | 0.5687 | ***0.0347*** |  |
|  |  |  |  |  |  |  |  |  |
|  | **Sin-11 Nu-** |  |  | 0.9449 | ***0.0012*** | ***0.0006*** | ***0.0014*** | 0.1770 |
|  | **Sin-11 Nu+** |  | 0.9449 |  | ***0.0003*** | ***0.0002*** | ***0.0003*** | ***0.0333*** |
| **Xyl** | **Gig-311 Nu-** |  | ***0.0012*** | ***0.0003*** |  | 0.9993 | 1.0000 | 0.1666 |
|  | **Gig-311 Nu+** |  | ***0.0006*** | ***0.0002*** | 0.9993 |  | 0.9980 | 0.0896 |
|  | **Sac-5 Nu-** |  | ***0.0014*** | ***0.0003*** | 1.0000 | 0.9980 |  | 0.1908 |
|  | **Sac-5 Nu+** |  | 0.1770 | ***0.0333*** | 0.1666 | 0.0896 | 0.1908 |  |
|  |  |  |  |  |  |  |  |  |
|  | **Sin-11 Nu-** |  |  | 0.7634 | 0.9068 | 0.4158 | 0.0896 | 0.6361 |
|  | **Sin-11 Nu+** |  | 0.7634 |  | 0.2258 | ***0.0421*** | ***0.0060*** | 0.0876 |
| **Man** | **Gig-311 Nu-** |  | 0.9068 | 0.2258 |  | 0.9372 | 0.4553 | 0.9937 |
|  | **Gig-311 Nu+** |  | 0.4158 | ***0.0421*** | 0.9372 |  | 0.9295 | 0.9989 |
|  | **Sac-5 Nu-** |  | 0.0896 | ***0.0060*** | 0.4553 | 0.9295 |  | 0.7701 |
|  | **Sac-5 Nu+** |  | 0.6361 | 0.0876 | 0.9937 | 0.9989 | 0.7701 |  |
|  |  |  |  |  |  |  |  |  |

# Supplementary Table S8

# – ANOVA cellulose content all samples.

#

|  |  |  | **All** |  |  |  |
| --- | --- | --- | --- | --- | --- | --- |
| **Effect** | **Sum of squares** | **Degrees of freedom** | **Mean square** | ***F*-ratio** | ***P*-value** | **Effect size (*η^2^*)** |
| Genotype | 242.06 | 2 | 121.03 | 4.95 | ***0.010*** | 0.112 |
| Nutrition level | 0.11 | 1 | 0.11 | 0.01 | 0.946 | 0.000 |
| Organ | 78.32 | 1 | 78.32 | 3.20 | 0.078 | 0.036 |
| Error | 1834.67 | 75 | 24.46 |  |  |  |
| **Total** | 2155.16 | 79 |  |  |  |  |
|  |  |  | **Leaf** |  |  |  |
| **Effect** | **Sum of squares** | **Degrees of freedom** | **Mean square** | ***F*-ratio** | ***P*-value** | **Effect size (*η^2^*)** |
| Genotype | 269.84 | 2 | 134.92 | 7.23 | ***0.002*** | 0.280 |
| Nutrition level | 11.71 | 1 | 11.71 | 0.63 | 0.434 | 0.012 |
| Geno. × Nut. Level. | 64.66 | 2 | 32.33 | 1.73 | 0.193 | 0.067 |
| Error | 616.05 | 33 | 18.67 |  |  |  |
| **Total** | 962.26 | 38 |  |  |  |  |
|  |  |  | **Stem** |  |  |  |
| **Effect** | **Sum of squares** | **Degrees of freedom** | **Mean square** | ***F*-ratio** | ***P*-value** | **Effect size (*η^2^*)** |
| Genotype | 57.51 | 2 | 28.75 | 1.16 | 0.324 | 0.051 |
| Nutrition level | 10.23 | 1 | 10.23 | 0.41 | 0.524 | 0.009 |
| Geno. × Nut. Level. | 194.61 | 2 | 97.30 | 3.94 | 0.029 | 0.173 |
| Error | 864.39 | 35 | 24.70 |  |  |  |
| **Total** | 1126.74 | 40 |  |  |  |  |

# Supplementary Table S9

– *P*-values for significant differences in cellulose content between genotypes attributed to nutrition level (Tukey test of the interaction Geno. × Nut. Level).

| **Leaf** | | | | | | |
| --- | --- | --- | --- | --- | --- | --- |
|  | **Sac-5 Nu-** | **Sac-5 Nu+** | **Gig-311 Nu-** | **Gig-311 Nu+** | **Sac-5 Nu-** | **Sac-5 Nu+** |
| **Sin-11 Nu-** |  | 0.785 | 0.636 | 0.140 | 0.928 | 1.000 |
| **Sin-11 Nu+** | 0.785 |  | 0.085 | ***0.009*** | 0.278 | 0.871 |
| **Gig-311 Nu-** | 0.636 | 0.085 |  | 0.894 | 0.994 | 0.519 |
| **Gig-311 Nu+** | 0.140 | ***0.009*** | 0.894 |  | 0.646 | 0.097 |
| **Sac-5 Nu-** | 0.928 | 0.278 | 0.994 | 0.646 |  | 0.863 |
| **Sac-5 Nu+** | 1.000 | 0.871 | 0.519 | 0.097 | 0.863 |  |
|  |  |  |  |  |  |  |
| **Stem** | | | | | | |
|  | **Sac-5 Nu-** | **Sac-5 Nu+** | **Gig-311 Nu-** | **Gig-311 Nu+** | **Sac-5 Nu-** | **Sac-5 Nu+** |
| **Sin-11 Nu-** |  | 0.742 | 1.000 | 1.000 | 0.206 | 1.000 |
| **Sin-11 Nu+** | 0.742 |  | 0.719 | 0.766 | 0.950 | 0.617 |
| **Gig-311 Nu-** | 1.000 | 0.719 |  | 1.000 | 0.179 | 1.000 |
| **Gig-311 Nu+** | 1.000 | 0.766 | 1.000 |  | 0.240 | 1.000 |
| **Sac-5 Nu-** | 0.206 | 0.950 | 0.179 | 0.240 |  | 0.136 |
| **Sac-5 Nu+** | 1.000 | 0.617 | 1.000 | 1.000 | 0.136 |  |

**Supplementary data Table S10** – ANOVA for acetyl bromide lignin determinations.

|  |  | **All** |  |  |  |  |
| --- | --- | --- | --- | --- | --- | --- |
| **Effect** | **Sum of squares** | **Degrees of freedom** | **Mean square** | ***F*-ratio** | ***P*-value** | **Effect size (*η^2^*)** |
| Genotype | 21.69 | 2 | 10.85 | 3.46 | *0.035* | 0.055 |
| Nutrition level | 2.40 | 1 | 2.40 | 0.77 | 0.384 | 0.006 |
| Organ | 39.70 | 1 | 39.70 | 12.67 | *0.001* | 0.100 |
| Error | 332.07 | 106 | 3.13 |  |  |  |
| **Total** | 395.86 | 110 |  |  |  |  |
|  |  | **Leaf** |  |  |  |  |
| **Effect** | **Sum of squares** | **Degrees of freedom** | **Mean square** | ***F*-ratio** | ***P*-value** | **Effect size (*η^2^*)** |
| Genotype | 13.25 | 2 | 6.63 | 1.58 | 0.216 | 0.062 |
| Nutrition level | 0.06 | 1 | 0.06 | 0.01 | 0.908 | 0.000 |
| Geno. × Nut. Level. | 4.34 | 2 | 2.17 | 0.52 | 0.599 | 0.020 |
| Error | 196.69 | 47 | 4.18 |  |  |  |
| **Total** | 214.34 | 52 |  |  |  |  |
|  |  | **Stem** |  |  |  |  |
| **Effect** | **Sum of squares** | **Degrees of freedom** | **Mean square** | ***F*-ratio** | ***P*-value** | **Effect size (*η^2^*)** |
| Genotype | 8.85 | 2 | 4.43 | 2.13 | 0.129 | 0.063 |
| Nutrition level | 2.70 | 1 | 2.70 | 1.30 | 0.259 | 0.019 |
| Geno. × Nut. Level. | 20.36 | 2 | 10.18 | 4.91 | 0.011 | 0.146 |
| Error | 107.85 | 52 | 2.07 |  |  |  |
| **Total** | 139.76 | 57 |  |  |  |  |

# Supplementary Table S11

– *P*-values for significant differences in lignin content between genotypes attributed to nutrition level (Tukey test of the interaction Geno. × Nut. Level).

| **Leaf** | | | | | | |
| --- | --- | --- | --- | --- | --- | --- |
|  | **Sin-11 Nu-** | **Sin-11 Nu+** | **Gig-311 Nu-** | **Gig-311 Nu+** | **Sac-5 Nu-** | **Sac-5 Nu+** |
| **Sin-11 Nu-** |  | 0.997 | 0.833 | 0.977 | 1.000 | 0.868 |
| **Sin-11 Nu+** | 0.997 |  | 0.569 | 0.838 | 0.970 | 0.610 |
| **Gig-311 Nu-** | 0.833 | 0.569 |  | 0.997 | 0.956 | 1.000 |
| **Gig-311 Nu+** | 0.977 | 0.838 | 0.997 |  | 0.999 | 0.999 |
| **Sac-5 Nu-** | 1.000 | 0.970 | 0.956 | 0.999 |  | 0.972 |
| **Sac-5 Nu+** | 0.868 | 0.610 | 1.000 | 0.999 | 0.972 |  |
|  |  |  |  |  |  |  |
| **Stem** | | | | | | |
|  | **Sin-11 Nu-** | **Sin-11 Nu+** | **Gig-311 Nu-** | **Gig-311 Nu+** | **Sac-5 Nu-** | **Sac-5 Nu+** |
| **Sin-11 Nu-** |  | 0.779 | 0.435 | 0.666 | ***0.008*** | 0.991 |
| **Sin-11 Nu+** | 0.779 |  | 0.995 | 1.000 | 0.222 | 0.975 |
| **Gig-311 Nu-** | 0.435 | 0.995 |  | 1.000 | 0.479 | 0.781 |
| **Gig-311 Nu+** | 0.666 | 1.000 | 1.000 |  | 0.315 | 0.933 |
| **Sac-5 Nu-** | ***0.008*** | 0.222 | 0.479 | 0.315 |  | ***0.032*** |
| **Sac-5 Nu+** | 0.991 | 0.975 | 0.781 | 0.933 | ***0.032*** |  |

# Supplementary Table S12

– Relative sugar release potential (%) based on saccharification results and dry biomass measures.

|  | Nu+ / 75% SWC |  | Nu- / 75% SWC |  | Nu+ / 15% SWC |  | Nu- / 15% SWC |
| --- | --- | --- | --- | --- | --- | --- | --- |
| Sin-11 | 111 |  | 121 |  | 33 |  | 61 |
| Sac-5 | 128 |  | 105 |  | 38 |  | 66 |
| Gig-311 | 100 |  | 94 |  | 33 |  | 55 |

The saccharification data (release of reducing sugars) was transformed to sugar release potential based on dry biomass measures. Sugar release for Gig-311 at Nu+ / 75% SWC was set to 100%.

# Supplementary Table S13

– Pearson’s correlation coefficient (r) between sugar release data and cell wall content.

| Pearson’s correlation coefficient | |
| --- | --- |
| Cell wall measure | Saccharification |
|  |  |
| Cryst. cellulose | -0.2 |
|  |  |
| Lignin | -0.6* |
|  |  |
| Arabinose | 0.7** |
| Galactose | 0.6** |
| Glucose | -0.2 |
| Xylose | 0.5 |
| Total matrix monosacch. | 0.4 |
| Ara/Xyl ratio | 0.6** |
| **P*<0.05; ** *P*<0.01 |  |
